# Nano-flow intracalvariosseous infusion enables skull-to-brain delivery of BBB-impermeable molecular and nanoscale agents in mice and rabbits

**DOI:** 10.7150/thno.138110

**Published:** 2026-08-24

**Authors:** Ji Hee Kang, O Hyun Lee, Min Suk Shim, Sehoon Kim, Dongyun Shin, Young Tag Ko

**Affiliations:** 1 College of Pharmacy and Gachon Institute of Pharmaceutical Sciences, Gachon University, Incheon, 21936, Republic of Korea.; 2 Division of Bioengineering, Incheon National University, Incheon 22012, Republic of Korea.; 3 Chemical & Biological Integrative Research Center, Korea Institute of Science and Technology, Seoul, 02792, Republic of Korea.; 4 KU-KIST Graduate School of Converging Science and Technology, Korea University, Seoul, 02841, Republic of Korea.

**Keywords:** skull-to-brain route, intracalvariosseous, blood-brain-barrier, sustained brain delivery, CNS drug delivery

## Abstract

**Rationale:**

The blood–brain barrier (BBB) remains a major obstacle to the delivery of therapeutics for central nervous system (CNS) diseases. Although several BBB-penetrating or BBB-bypassing strategies have been investigated, there remains a need for sustained, controllable delivery routes that can support prolonged CNS exposure of BBB-impermeable agents. Recent anatomical studies have shown direct connections between skull bone marrow and brain, suggesting that the skull may serve as an alternative access route to the brain. Here, we assessed the feasibility of sustained intracalvariosseous infusion (ICO) as skull-to-brain delivery approach for BBB-impermeable molecular and nanoscale agents in mice and rabbits.

**Methods:**

Four-week ICO was performed using a species-adapted nano-flow pump–cannula configuration that positioned the cannula terminus within the skull diploic space. A positive comparator was established by positioning the terminus beyond the inner skull cortex. Placement was verified by conventional CT and micro-CT. Brain-associated exposure of BBB-impermeable paclitaxel, antisense oligonucleotides and gold nanoparticles was assessed, along with systemic toxicity, bone marrow-derived immune activation, and neuroinflammation.

**Results:**

ICO produced measurable brain-associated exposure of all three model agents in both species. Relative to the positive comparator, ICO achieved the highest relative brain-associated exposure for ASO, reaching approximately 24–28% of comparator levels, whereas PTX and AuNP showed lower but detectable exposure. Four-week ICO was not associated with overt hematological, biochemical, histopathological, or neuroinflammatory abnormalities under the present experimental conditions.

**Conclusions:**

These findings support the technical feasibility of ICO-based sustained infusion as a skull-to-brain delivery platform for controlled and prolonged CNS exposure of BBB-impermeable molecular and nanoscale agents through an extracerebral skull compartment.

## Introduction

Overcoming the blood–brain barrier (BBB) remains a central challenge in the development of effective therapies for central nervous system (CNS) disorders 1, 2. Clinically and preclinically, three main strategies have been explored: (1) enhancing the BBB permeability by chemical structure modification or carrier loading; (2) transiently opening the BBB by osmotic agents or external stimuli; and (3) bypassing the BBB via parenchymal, intracerebroventricular (ICV), intrathecal (IT) and intranasal (IN) administration 1, 3-5. Despite significant advances, these approaches face limitations in widespread clinical application. Various BBB penetrating carrier-based approaches have demonstrated promising efficacy in preclinical studies, but their clinical translation remains challenging due to safety, efficacy, reproducibility and related other issues 5-7. Transient BBB opening approach provides the opportunity to deliver drugs previously excluded by the BBB, but further improvements in safety, spatial control, and dosing reproducibility are still needed for broader clinical implementation 8, 9. Parenchymal and ICV administration can achieve high local concentrations but require direct access to brain or cerebrospinal fluids (CSF) compartments, carrying invasive procedure-related risks and complications 10-12. IN administration involving application to nasal mucosa is non-invasive and convenient but it is limited by variable deposition, mucociliary clearance, and challenges in reproducible dosing 13-15. These limitations highlight the need for alternative CNS access strategies that can provide sustained, controllable, and anatomically localized exposure of BBB-impermeable agents.

Recent anatomical studies have shown direct connections between skull bone marrow, meninges, and brain-associated compartments 16, 17, suggesting that the skull may serve as a previously underexplored access route to the CNS 18. These skull-meninges-brain connections have been shown to support immune-cell trafficking and direct access to CSF 16, 17, 19-26, providing an anatomical rationale for exploring the skull as an extracerebral access route to brain-associated compartments. Inspired by these new findings, our prior proof-of-concept study demonstrated that intracalvariosseous administration (ICO)—direct application into the diploic space between the outer and inner cortex of skull bone—can increase brain exposure of selective CNS drugs by approximately one to two orders of magnitude compared with intravenous (IV) administration, supporting the feasibility of a skull-based brain access strategy 18. However, that initial study was limited by its short-term administration design, mouse-only scale, comparison primarily with systemic delivery, and focus on small molecules. In particular, the thin mouse calvarium with ~0.3 mm thickness 27 and limited diploic space restrict the anatomical and surgical relevance of mouse-only ICO studies for human translation (~6 mm thick skull bone) 28. Moreover, studies with larger molecules and nanoparticles other than small molecules could provide broad applicability and versatility of the skull to brain route.

Here, we aim to advance ICO from a short-term administration concept to a four-week skull-to-brain infusion paradigm and to assess its feasibility beyond the rodent skull in a translationally relevant intermediate animal model. We configured infusion pump implant with nano-flow rate (nanoIPI) by integrating a commercially available nano-flow osmotic pump with species-specific customized guide cannulae, allowing the infusion terminus to be positioned within the skull diploic space in mice and rabbits. Using subcalvariosseous administration (SCO), in which the cannula terminus is positioned beyond the inner skull cortex, as a positive comparator, we evaluated whether sustained ICO can support brain exposure of BBB-impermeable agents spanning a small molecule, an oligonucleotide, and a nanoparticle. Cannula placement was confirmed by conventional computed tomography (CT) and micro-CT scans. Brain exposure and regional brain distribution of model agents after ICO were determined by quantitative analysis including LC-MS/MS, gel electrophoresis and ICP-MS. Finally, systemic tolerability and neuroinflammatory responses were assessed to determine the feasibility of prolonged ICO.

## Materials and Methods

### Materials

Paclitaxel (PTX) was purchased from TCI (Tokyo, Japan). Biotinylated scramble antisense oligonucleotide (ASO) was synthesized by Bioneer (Daejeon, Korea). Gold nanoparticle (AuroVist™, 15 nm) was purchased from Nanoprobes, Inc (NY, USA). Detailed information of PTX, ASO and AuNP are summarized in **Figure S1**. Cy3-labeled AuNP was purchased from SIGMA Aldrich (MO, USA). Streptavidin-coated magnetic beads (Dynabeads™ M-270 Streptavidin, Invitrogen™, MA, USA) were purchased from Invitrogen™ (MA, USA). D-biotin and formic acids were purchased from TCI (Tokyo, Japan). LC-MS/MS-grade acetonitrile, ethyl acetate, and water were obtained from J.T. Baker (PA, USA). Analytical grade 60% nitric acid was purchased from DUKSAN (Seoul, Korea). Male BALB/c mice were obtained from Orient Bio (Seongnam, Republic of Korea). Male New Zealand white rabbits were obtained from Hanlim experimental animal Co. (Hwasung, Korea). Other buffers and analytical-grade chemicals were obtained from J.T. Baker (PA, USA), TCI (Tokyo, Japan) and SIGMA Aldrich (MO, USA).

### Study design

**Figure 1:**
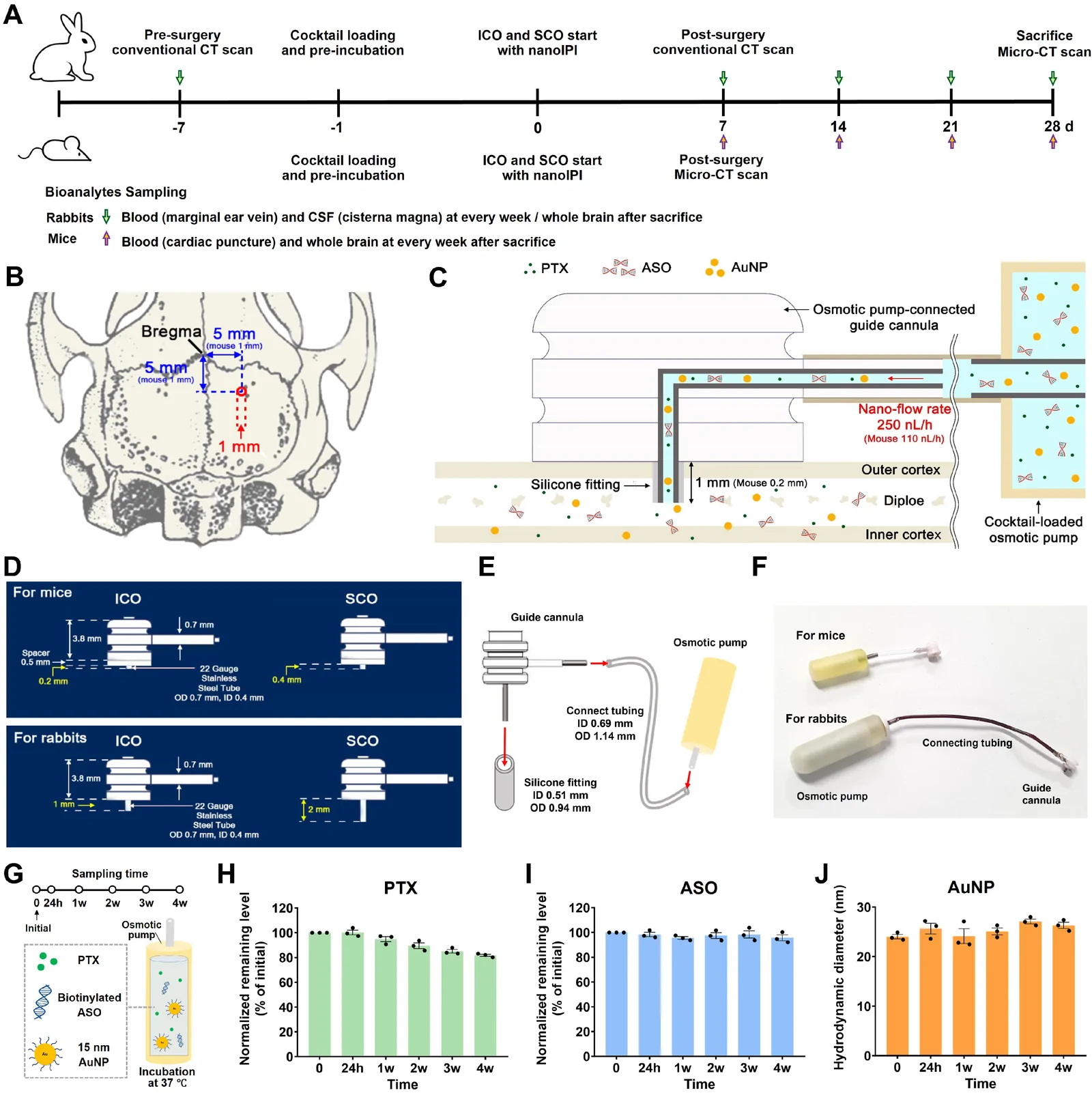
** Four-week ICO infusion design and species-adapted pump–cannula configuration. (A)** Schematic overview of the four-week ICO or SCO infusion study in mice and rabbits. **(B)** Dorsal view of the rabbit skull showing the target site for guide cannula placement in the right parietal bone. The red circle indicates the skull drilling site. The parentheses indicate the numerical values for the thinning areas in mice skulls. **(C)** Coronal schematic showing the ICO cannula terminus positioned within the skull diploic space. In rabbits, the cannula terminus was positioned within the diploic space at a depth of 1 mm, and a cocktail containing PTX, ASO, and AuNP was infused at a nominal flow rate of 250 nL/h for four weeks. In mice, the cannula terminus was positioned within the diploic space at a depth of 0.2 mm, and the cocktail was infused at 110 nL/h for four weeks. **(D)** Species-adapted customized guide cannulae for ICO and SCO in mice and rabbits. The stainless-steel tube length was customized according to skull thickness and intended cannula terminus position. **(E)** Assembly of the nanoIPI. Silicone fitting was applied over the stainless-steel tube to seal the interface between the guide cannula and skull hole and to minimize leakage. The osmotic pump was loaded with the cocktail and connected to the guide cannula via medical-grade tubing. **(F)** Assembled photo image of the nanoIPI consisting of a guide cannula with silicone fitting, connecting tubing and nano-flow osmotic pump. **(G)** Schematic illustration of the stability assessment of the model-agent cocktail under pump-relevant conditions. The formulation containing PTX, biotinylated ASO, and AuNP was loaded into an osmotic pump reservoir and incubated at 37 °C. Aliquots were collected at the initial time point, 24 h, and 1, 2, 3, and 4 weeks for agent-specific stability analyses. **(H)** PTX, **(I)** ASO and **(J)** AuNP stability results over the four-week incubation period. The remaining PTX and ASO level at each time point was normalized to the initial time point. AuNP colloidal stability was evaluated by DLS-based hydrodynamic diameter. Data are presented as mean ± SEM (n = 3).

The experimental design is presented in **Figure 1A**. This study was designed as a four-week sustained infusion study to evaluate whether ICO can maintain skull-to-brain exposure of BBB-impermeable model agents in mice and rabbits. Subcalvariosseous administration (SCO) was used as a positive comparator in which the cannula terminus was positioned beyond the inner skull cortex and beneath the skull, thereby providing an upper-bound comparator for estimating relative ICO-mediated passage across the skull–meningeal interface. A pre-surgery conventional CT scan (Alexion™ TSX-032A, Toshiba Medical Systems Corp., Ohtawara Japan) was performed a week before initiating the study to measure the thickness of the skull in each rabbit while under anesthesia. A post-surgical conventional CT scan was performed one week after surgery to verify anatomical placement of the pump-cannula configuration in rabbits. To obtain high-resolution images related to the skull after nanoIPI installation and ICO or SCO with nanoIPI, a micro-CT scan (vivaCT 80, SCANCO Medical, Switzerland) was carried out with the dissected whole skull bone after sacrifice. Similarly, a micro-CT scan was carried out in live mice a week after surgery to check the successful installation of ICO and SCO with the nanoIPI.

In rabbits, serial blood and CSF samples were collected weekly, and terminal brain tissues were collected at week 4. In mice, terminal brain tissues were collected weekly from independent cohorts to determine time-dependent brain exposure. Therefore, rabbit CSF AUC and mouse brain AUC were analyzed as complementary but non-equivalent indices of CNS exposure.

### Animals

The animal experiments were performed under protocols approved by the respective Institutional Animal Care and Use Committees (IACUCs) and were reported in accordance with ARRIVE guidelines. Only male animals were used to reduce variability associated with sex-dependent anatomical and pharmacokinetic factors in this initial feasibility study. This limitation is acknowledged in the Discussion. Each animal was considered an independent biological replicate. Group sizes are indicated in each figure legend and table.

### Mice

Experimental protocols were approved by the IACUCs at Gachon University (Republic of Korea) and were conducted in compliance with the Animal Ethics Committee guidelines (Approval number: GUI-2022-IA0057-00). Male BALB/c mice, five weeks old and weighing 20–25 g, were used. A total of 32 mice were included in the study. Each week for four consecutive weeks, eight mice were randomly allocated to the ICO and SCO groups (n = 4 per group). Randomization was performed using a simple random assignment strategy. Sample sizes were selected based on previous pilot experiments and feasibility considerations for this initial cross-species delivery study. No formal power calculation was performed. Blinding was not applied during surgery or imaging-based placement confirmation, or bioanalytical analysis.

### Rabbit

Experiments were conducted according to the guidelines of the Ethics Committee for the Protection and Use of Experimental Animals of KNOTUS Co., Ltd (currently HLB BioStep Co., Ltd) (Incheon, Korea) [Approval numbers: 23-KE-0101 and 23-KE-0273]. Nine male white New Zealand rabbits, five months old and weighing 3.0–3.5 kg, were used. Animals were randomly divided into the ICO (n = 5) and SCO (n = 4) groups. The number of animals was determined based on previous experience and feasibility, in accordance with ARRIVE guidelines, to minimize animal use while ensuring meaningful results.

### Cocktail preparation

To evaluate the brain drug delivery of small molecules, nucleic acids, and nanoparticles after ICO or SCO with nanoIPI, we prepared a cocktail with PTX, ASO, and AuNP. The physicochemical characteristics of the three model drugs were described in **Figure S1**. The cocktail was used to compare the skull-to-brain passage of model agents with distinct physicochemical properties under the same sustained infusion condition, rather than to evaluate pharmacological interactions among the three agents. The ASO was a phosphorothioate-modified, biotinylated scramble oligonucleotide used as a bioanalytical surrogate for nucleic acid therapeutics. It was not designed to induce target knockdown or therapeutic pharmacodynamic effects. PTX was dissolved in Cremophor EL:ethanol (1:1, v/v) at a concentration of 12.5 mg/mL. ASO was dissolved in distilled water at a concentration of 1 nmol/μL. AuNP was dissolved in PBS at a concentration of 200 mg/mL. PTX, ASO and AuNP were diluted with PBS and mixed to prepare a cocktail formulation at a dose for rabbits (PTX, 0.15 mg/kg; ASO, 30 nmol/kg; AuNP 1 mg/kg) in a final volume of 250 μL. For mice, PTX, ASO, and AuNP were diluted with PBS and mixed at a dose (PTX, 5 mg/kg; ASO, 2 μmol/kg; AuNP 25 mg/kg) in a final volume of 100 μL. The ethanol in the cocktail evaporated under vacuum overnight. Doses and infusion volumes were selected based on species-specific pump capacity, skull dimensions, and analytical detectability in pilot experiments. The final cocktail was visually inspected for precipitation or visible aggregation before pump loading.

### Species-adapted infusion pump implant with nano-flow rate (nanoIPI)

The nano-flow osmotic pump was commercially available. The technical configuration in this study consisted of adapting the pump to ICO or SCO by using species-specific customized guide cannulae, silicone fittings, and connecting tubing (**Figure 1E**). The customized guide cannula (22 gauge, P1 Technologies) and osmotic pump (110 nL/h for mice, 250 nL/h for rabbits, Alzet^®^) were purchased. The cannula depth was selected to position the terminus either within the diploic space for ICO or beyond the inner skull cortex for SCO, based on species-specific skull thickness. For mice, the length of the stainless-steel tube in the guide cannula was customized and adjusted with a spacer (0.5 mm) to 0.2 mm or 0.4 mm for ICO or SCO installations, respectively. For rabbits, the length of stainless-steel tube was customized to be 1 mm or 2 mm for ICO or SCO installation, respectively (**Figure 1D**). As shown in **Figure 1E,** the stainless-steel tube of the guide cannula was fitted with silicone tubing (standard silicone tubing, 0.51 mm ID, 0.94 mm OD, HelixMark^®^). As shown **Figure 1E and 1F**, the osmotic pump was loaded with the cocktail and then connected to the guide cannula via connecting tubing (1.5 cm for mice, 5 cm for rabbits, medical-grade polyvinylchloride, 0.69 mm ID, 1.14 mm OD, Alzet^®^). The fully assembled pump–cannula configuration was pre-incubated in sterile saline at 37 °C for 24 h to initiate pump priming before implantation.

### Stability assessment of model agents under pump-relevant conditions

To evaluate the stability of the model agents during the four-week continuous infusion period, PTX, ASO, and AuNP were incubated under pump-relevant conditions. The model-agent formulation was prepared using the same vehicle and concentration as those used for *in vivo* infusion (mouse) and incubated at 37 °C. Samples were collected at predefined time points, including 24 h and 4 weeks, and analyzed using agent-specific methods. Freshly prepared formulation was used as the initial reference sample. PTX stability was assessed by LC-MS/MS and the remaining PTX percentage was calculated by normalizing the measured concentration at each time point to that of the initial formulation. ASO integrity was evaluated by gel electrophoresis. The percentage of remaining intact ASO was calculated by normalizing the intact-band intensity at each time point to that of the initial formulation. AuNP colloidal stability was assessed by dynamic light scattering (DLS) (ELSZ-1000, Otsuka Electronics, Osaka, Japan) and UV-vis spectroscopy (Cary 60 UV-Vis, Agilent, CA, USA). At each time point, AuNP-containing samples were diluted in the PBS and analyzed for hydrodynamic diameter and polydispersity index (PDI) using DLS. UV-vis absorbance spectra were obtained over the wavelength range (400 nm – 800 nm). Changes in hydrodynamic diameter, PDI, absorbance intensity, peak broadening, or shifts in the maximum absorbance wavelength were used to evaluate AuNP aggregation or destabilization during incubation. These stability assays were intended to determine whether each model agent retained analytical integrity or colloidal stability over the four-week infusion-relevant period, rather than to fully reproduce *in vivo* recovery from the implanted pump system.

### Animal surgery and nanoIPI installation

#### Mice

To perform ICO and SCO with nanoIPI in mice, the surgical procedure was designed to distinguish infusion into the diploic space from infusion after removal of the inner skull cortex. For ICO, the right parietal bone was drilled to a depth of approximately 200 μm, allowing access to the diploic space while preserving the inner skull cortex. For SCO, drilling was extended through the skull until the inner cortex was removed and the dura was visually exposed, thereby allowing the cannula terminus to be positioned beneath the skull as a positive comparator.

The mice were first anesthetized with isoflurane (1 – 1.5% in N_2_O:O_2_ [70:30 vol%]) and maintained at a core temperature of 37 °C. After anesthesia, the scalp was incised and the fascia was removed to expose the skull. To create space for the osmotic pump of the nanoIPI, the dorsal subcutaneous space was separated using forceps and the osmotic pump connected to the guide cannula, was carefully positioned in this space. A hole was created in the right parietal bone at a stereotaxic location 1.0 mm posterior and 1.0 mm lateral to bregma. The drilling depth and cannula projection were selected together to position the cannula terminus within the diploic space for ICO or beyond the inner skull cortex for SCO. This drilling was performed using an inverted corn-shaped bit with a diameter of 1 mm, utilizing the Auto-speed Craniotomy-Shape mode and a robot stereotaxic system equipped with StereoDrive software (NeuroStar, Tubingen, Germany). Customized guide cannula was securely installed in the drilled area of the skull using cyanoacrylate gel glue and resin-modified glass ionomer cement. Finally, the wound was closed with sutures.

#### Rabbits

To perform the surgical procedure for ICO and SCO with nanoIPI in the rabbits, the following procedure was carried out (**Figure S3**). Rabbit skull thickness was measured preoperatively by CT to guide individual depth selection and reduce the risk of unintended penetration during ICO installation. The rabbits were initially anesthetized with an intramuscular injection of 5 mg/kg tiletamine-zolazepam (Zoletil® 50, Virbac, Seoul, Korea) and 2 mg/kg xylazine (Rompun®, Bayer AG, Leverkusen, Germany). After the rabbit was placed in the ventral recumbent position, the surgical site was shaved, disinfected, and draped. A midline skin incision was made from the middle of the skull toward the occipital protuberance, followed by deep fascia incision and periosteal stripping to expose the skull.

The right parietal bone was drilled at a position 5.0 mm lateral and 5.0 mm caudal to bregma using a hand drill (FOREDOM, K.1070) with an inverted cone-shaped bit with a diameter of 1 mm (**Figure 1B**). For ICO, drilling was performed to a depth of 1 mm, allowing the cannula terminus to remain within the diploic space while preserving the inner skull cortex. For SCO, drilling was extended through the inner skull cortex until the dura was visually exposed; the cannula terminus was then positioned at the exposed dural surface as a positive comparator. Although a nominal drilling depth of approximately 2 mm was used for SCO based on preoperative CT-based thickness, dural exposure rather than fixed depth alone was used as the surgical endpoint.

The subcutaneous layer of the right side of the neck was separated by forceps to secure a space for the osmotic pump, which was then placed in the space obliquely to the right. The guide cannula was securely installed in the drilled area of the skull with cyanoacrylate gel glue (Loctite 454) and resin-modified glass ionomer cement (GC FujiCEM^®^2) (**Figure 1C**). The wound was subsequently closed using sutures and surgical staples. A neck collar was used to protect the implanted pump–cannula system during the postoperative period.

### CT and micro-CT-based anatomical validation of nanoIPI placement

A week before and after the surgery to install the ICO or SCO, the rabbits were anesthetized and evaluated by computed tomography (CT; Alexion™ TSX-032A, Toshiba Medical Systems Corp., Ohtawara Japan) using the following settings: 120 kVp, 100 mA, 1 mm slice thickness, 0.5 mm slice interval, 0.75 s speed, single axial tomogram. Images of the CT scans were analyzed using INFINITT CD Viewer (INFINITT Healthcare Co., Ltd., Seoul, South Korea).

Live mice and dissected rabbit skulls were scanned with a Viva CT 80 (Scanco Medical, Bassersdorf, Switzerland) at the Micro-CT Core Laboratory at HLB BioStep Co., Ltd. (Incheon, Korea). Each specimen was horizontally oriented and spaced apart using soft sponges. The holders were positioned vertically on the turntable of the micro-CT system. The following settings were selected: 45 kVp, 117 μA X-ray intensity, 79.9 mm FOV/diameter, and 78 μm voxel size. The 3D rendering of the skull was shown using the microCT Evaluation Program V6.6 (Sanco Medical software). RadiAnt DICOM Viewer Program (Medixant, Poznan, Poland) was used to display 3D multiplanar reconstruction (MPR) images and to analyze the data. Quantitative catheter placement analysis was performed using CT and micro-CT images. The measured cannula depth was defined as the distance from the outer skull surface at the drilling site to the cannula terminus. For ICO, the measured depth was compared with the intended target depth, and preservation of the inner skull cortex was assessed. For SCO, successful placement was defined as full-thickness skull penetration with dural exposure. Placement success rate and deviation from the intended target depth were calculated for each group.

### *Ex vivo* confocal imaging of mouse skull- brain sections

To investigate distribution of Cy3-labeled AuNP in the skull diploic space and adjacent brain-associated regions after ICO, we performed *ex vivo* skull-brain imaging using a laser scanning confocal microscope (A1 plus, Nikon, Japan) in mice. A pump–cannula implant loaded with Cy3-labeled AuNP was installed for ICO in mice. After a week, to identify skull bone, mice were injected with IVISense Osteo 680 Fluorescent Probe (OsteoSense™, 4 nmole/mouse, Revvity) via tail vein one day before imaging. The vessels were labeled *in vivo* with ABflo® 488 Rabbit anti-Mouse CD31 mAb (A23701, ABclonal, MA, USA) of 100 μL through tail vein injection 1h before sacrifice. Mice were transcardially perfused with 20 mL PBS to remove intravascular contents before tissue collection. The skull with brain was dissected, cleaned and fixed with 4% paraformaldehyde in PBS overnight, then washed twice in PBS for 10 min. The skull with brain was embedded in optimal cutting temperature (OCT) compound (FSC 22® Clear, Leica, Germany) on dry ice and kept at -80 °C. The skull with brain was later coronally sectioned on a cryostat (CM1850, Leica, Germany). Z-stack images were acquired with 0.5 μm steps at 10X magnification with a resolution of 512 by 512 pixels. Images were analyzed using NIS-E software (Nikon, Japan).

### Bioanalytes sampling

Blood samples (1 mL) were collected from the marginal ear vein of the rabbits and via cardiac punctures in the mice each week after ICO or SCO installations. Immediate centrifugation of the blood aliquots at 10,000 rpm and 4 °C for 10 min was performed to recover plasma. CSF samples (1 mL) were collected from the cisterna magna of the rabbits. CSF samples were visually inspected for blood contamination before processing. In the case of rabbits, the brain was collected four weeks after administration and homogenized using a glass homogenizer after the addition of PBS equating to the weight of each tissue sample. For mice, animals were euthanized in a CO_2_ chamber at predetermined time points after ICO or SCO, and then transcardially perfused with 20 mL of PBS. Effect of perfusion was assessed using predefined gross and microscopic quality-control criteria, including visual blanching of the liver and brain surface and clearance of blood from the perfusion effluent. In addition, hematoxylin and eosin (H&E)-stained brain sections were examined to assess residual intravascular erythrocytes within cortical microvessels (**Figure S15**). H&E imaging analysis was done by using slide scan (Pannoramic scan II digital scanner, 3DHISTECH, Budapest, Hungary) at Core-facility for Cell to In-vivo imaging. The skull was longitudinally opened from the occipital to nasal region, taking care to preserve the integrity of the brain 29. The brain was then carefully removed using forceps without scraping or disturbing the inner skull surface. Visible dura mater and meningeal tissues attached to the cortical surface were gently removed using fine forceps before regional brain dissection. Subsequently, the cerebral cortex, subcortex, hippocampus, and cerebellum were dissected from each hemisphere using fine forceps and curved microtweezers 30. To isolate dural meninges within the skull, its edges were gently scraped with precision forceps, ensuring its careful separation from the inner surface of the skull. Each brain sample was homogenized in a volume of PBS corresponding to the tissue weight using Bioprep-24 homogenizer (Bioand, Seoul, Korea). All biological samples (plasma, CSF, and brain homogenates) were stored at -80 °C until the time of quantification analysis.

### PTX quantification by LC-MS/MS

To quantify the PTX in plasma, CSF, and whole brain homogenate samples, LC-MS/MS analysis was performed according to a previously established method after liquid-liquid extraction (LLE) 31. Plasma samples (50 μL), CSF (50 μL), or brain homogenates (100 μL) were combined with containing internal standard-containing mobile phase (10 μL), followed by the addition of ethyl acetate (250 μL). The mixture was vortexed for 1 min and centrifuged at 10,000 rpm and 4 °C for 10 min. The resulting organic layer was collected and evaporated to dryness under vacuum. The evaporated residues were reconstituted in a mobile phase (20 μL) and immediately analyzed using LC-MS/MS (6490 QQQ mass spectrometer coupled with an LC 1100 series, Agilent Technologies, CA, USA). Separation of the chromatographic compounds was performed using a Sepax BR-C18 (5 μm, 1.0 × 100 mm) analytical column at 45 ℃. PTX was quantified in positive MRM mode by monitoring the precursor-to-product ion transition at 876.3/308.0 (collision energy 20 eV). The analyte was separated via isocratic elution with a mixture of acetonitrile and water containing 0.1% formic acid (60:40, v/v). The flow rate was maintained at 0.10 mL/min throughout the 5 min run time.

### ASO quantification by gel electrophoresis

Because ASO quantification was based on streptavidin-mediated recovery and gel densitometry, the method was intended to detect intact biotinylated ASO rather than total ASO metabolites. Known amounts of biotinylated ASO spiked into blank plasma, CSF, skull, dura or brain homogenate were processed in parallel to generate matrix-matched calibration curves. Streptavidin-coated magnetic beads of 30 μL (Dynabeads™ M-270 Streptavidin) were pre-washed thrice with 1X Binding and washing (B&W) Buffer (0.5 mM EDTA, 1 M NaCl, 5 mM Tris-HCl, 0.05% tween 20, pH 7.5) and were incubated with the prepared plasma and brain homogenate solutions at 37 ℃ for 2 h. The beads were washed with 1X B&W Buffer, PBS and 25 mM NaOH. Biotinylated ASO were eluted by incubation with D-biotin (1 mM in distilled water) at 95 ℃ for 20 min. Eluates were subjected to 2% agarose gel electrophoresis and imaging (Image lab, Bio-Rad, CA, USA). The gel bands were analyzed using Image J software (a program developed by the National Institutes of Health and accessible online, https://imagej.nih.gov/ij/). Matrix-matched calibration curves were generated by spiking known amounts of biotinylated ASO into blank rabbit brain, mouse brain, mouse skull, or mouse dura homogenates, followed by the same streptavidin bead-based extraction and gel densitometry procedure used for experimental samples. Linear regression was performed over the detectable range of 20–100 pmol. The 10-pmol standard was below the detection limit under the present assay conditions and was excluded from regression analysis (**Figure S7**). Extraction recovery and intra-assay precision were evaluated using a representative mid-level quality-control (QC) sample containing 50 pmol of biotinylated ASO. For each tissue matrix, four independent matrix-matched extraction replicates were processed using the same streptavidin bead-based recovery and gel densitometry procedure as experimental samples. Recovery was calculated by comparing the corrected band intensity of extracted samples with that of non-extracted 50 pmol ASO standards, and intra-assay precision was expressed as the coefficient of variation (%CV).

### AuNP quantification by ICP-MS

The brain homogenate samples of 1 mL were digested in 60% nitric acid of 4 mL at 90 ℃ for 8 h. Leftover undissolved particles were filtered using a PVDF membrane syringe filter with 0.45 μm pore size. The filtrate of 1 mL was then diluted to 10 mL with distilled water containing 2% HCl and transferred to 15 mL polypropylene tubes. Plasma and CSF samples were digested and diluted using the same acid digestion procedure, with sample volumes adjusted according to availability. Total gold concentration, used as a surrogate for AuNP-associated tissue exposure, was analyzed by ICP-MS (iCAP Q, Thermo, MA, USA). Free nitric acid for pure blank, blank tissue samples, nanoparticle standards without brain homogenates for positive control calibration curve, and elemental standards were prepared and analyzed concurrently with test samples.

### Tissue processing and immunohistochemistry

Skulls and brain tissues were immersion-fixed in 4% paraformaldehyde overnight at 4 °C. Skull bones were decalcified in CUBIC-B reagent (TCI, Japan) for 5 days at 37 °C with gentle agitation (50 rpm). Brains were cryoprotected by immersion in 30% sucrose (w/w in PBS) until they equilibrated and sank, and then embedded in OCT compound on dry ice, and kept at -80 °C. Coronal brain sections (20 μm) were then cut on a cryostat and mounted onto Superfrost Plus slides (Thermo Fisher Scientific, USA). Skulls or brain sections underwent antigen retrieval by incubation with proteinase K (15 μg/mL in PBS) for 30 min, followed by blocking of endogenous peroxidase activity with 3% H_2_O_2_ for 10 min. Then sections were blocked and permeabilized for 30 min in blocking buffer (PBS containing 0.2% Triton X-100 and 5% goat serum). Primary antibodies (Mouse anti-Mouse Iba1 [1:200; ab283319, abcam], Rabbit anti-Mouse IL-1β [1:200; ab283818, abcam], Rabbit anti-Mouse CD68 [1:200; ab283654, abcam]) diluted in PBS containing 1% BSA were applied to sections overnight 4 °C. Following three washes in PBS, sections were incubated for 1 h at room temperature with secondary antibodies (Alexa Fluor® 488-Goat anti-Mouse IgG [1:500; ab150113, abcam], Alexa Fluor® 555-Goat anti-Rabbit IgG [1:500; ab150078, abcam]) diluted in PBS containing 1% BSA and 30% glycerol. Sections were then washed in PBS, counterstained for 30 min with DAPI (5 μg/ml; Thermo Fisher Scientific, USA) for brain and OsteoSense™ (10 nmol/mL; Revvity, USA) for skull, followed by a final PBS wash. The skulls were cleared in CUBIC-R reagent (TCI, Japan) for 30 min before mounting on slide glass. Coverslips were applied using Fluoromount™ aqueous mounting medium (SIGMA, USA). Images were then acquired by confocal microscopy (Nikon, Japan). Quantitative analysis of immunofluorescence images was performed using NIS-Elements (Nikon, Japan). For each animal, three-predefined regions of interest (ROIs) were analyzed in comparable ipsilateral and contralateral regions. In skull whole-mount samples, ROIs were selected from the ipsilateral drilling site and the corresponding contralateral non-drilled region. Mean fluorescence intensities were measured after background subtraction and Manders’ overlap coefficients were calculated for co-localization analysis.

### Complete blood count and biochemical analysis

Complete blood count (CBC) and biochemical analysis were tested using blood samples obtained from mice and rabbits at four weeks after ICO and SCO. Serum biochemical metrics, such as alanine aminotransferase (ALT), alkaline phosphatase (ALP), aspartate aminotransferase (AST), blood urea nitrogen (BUN), albumin, and creatinine in the blood were assessed through HLB BioStep company (Incheon, Republic of Korea).

### Data analysis

To evaluate the pharmacokinetic outcomes of the PTX, ASO, and AuNP based on the route of administration (ICO or SCO), the concentrations of molecules in plasma and CSF samples were graphed over time. The area under the curve in plasma concentration-time profiles (AUC_plasma_), whole brain concentration-time profiles (AUC_brain_), and CSF concentration-time profiles (AUC_CSF_) were determined by non-compartmental analysis using Phoenix WinNonlin 6.4 (Pharsight, Mountain View, CA). In case of the rabbits, the drug concentration in the whole brain after four weeks post administration (C_brain_) was corrected for the plasma volume of the brain using the equation:



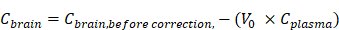



where, *C_brain,before correction_* = brain concentration (ng/g) after four weeks post administration, *V_0_* = plasma volume of the brain (μL/g), and *C_plasma_* = plasma concentration (ng/mL) after four weeks post administration. The following *V_0_* value was chosen as 23.1 ± 9.0 μL/g 32 for rabbits. For ASO, plasma concentrations were below the detection limit and no plasma-volume correction was applied.

ICO-mediated brain exposure was expressed as an ICO/SCO exposure ratio. For AUC-based comparisons, this ratio was termed relative brain availability (F_brain_ for mouse brain AUC or F_CSF_ for rabbit CSF AUC). For terminal brain concentrations at week 4, the ratio was termed K_brain,4weeks_. These ratios are analogous in form to systemic bioavailability calculations but should not be interpreted as absolute bioavailability because SCO is a positive comparator rather than a true 100% reference. The relative brain availability and the terminal ICO/SCO brain concentration ratio using the equation:



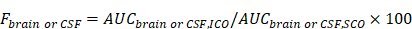





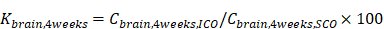



These values were interpreted as relative exposure indicators rather than independent inferential endpoints. Therefore, formal hypothesis testing was not performed for these ratio values. Measurement precision was indicated by the corresponding AUC_CSF_, AUC_brain_, or C_brain_ values reported as mean ± SEM.

### Statistical analysis

Data are expressed as mean ± SEM. Each animal was considered an independent biological replicate. Exact n values are indicated in the figure legends. No animals were excluded from analysis. Predefined exclusion criteria included technical failure of pump installation or sample collection. Two group comparisons were performed using unpaired two-tailed Student’s T-tests. Multiple-group comparisons were performed using one-way ANOVA followed by Tukey’s multiple-comparison test through GraphPad software. Statistical significance is marked by * and ** (# and ##), indicating p < 0.05 and p < 0.01.

## Results

### Sustained ICO infusion design in mice and rabbits

We designed a four-week pump-mediated ICO study in mice and rabbits to evaluate sustained skull-to-brain delivery across species (**Figure 1A**). ICO and subcalvariosseous administration (SCO) were applied to the right parietal bone using a nano-flow pump-cannula configuration load with a cocktail of PTX, ASO and AuNP (**Figure 1B – 1F; Figure S1**). In ICO, the cannula terminus was designed to remain within the skull diploic space, whereas in SCO, the terminus was positioned beyond the inner skull cortex. SCO was used as a positive comparator, not as an absolute 100% brain-availability reference. The infusion setup consisted of a commercially available nano-flow osmotic pump connected to a species-adapted customized guide cannula through medical-grade tubing. The stainless-steel tube in the guide cannula was customized as ICO and SCO for mice and rabbits (**Figure 1D**). Silicone fitting was applied over the stainless-steel for a tight closure between the guide cannula and the circumferential surface of the skull hole (**Figure 1E**). Before *in vivo* infusion, the physicochemical properties of the three model agents were characterized to confirm their suitability as representative BBB-impermeable molecular and nanoscale agents. PTX was selected as a hydrophobic small-molecule drug, the phosphorothioate 20-base pair ASO as a macromolecular nucleic acid, and 15-nm AuNP as a nanoparticle agent 33-35. Their analytical identity, detection methods, and baseline physicochemical properties are summarized in Fig. S1 and described in the Methods. To validate the integrity of the agents during the four-week continuous infusion, we further assessed the stability of the cocktail under pump-relevant conditions. The cocktail was loaded into an osmotic pump reservoir and incubated at 37 °C, with aliquots collected at the initial time point (0), 24 h, and 1, 2, 3, and 4 weeks (**Figure 1G**). Under these pump-relevant conditions, both PTX and intact ASO levels retained more than 80% of their initial levels over the four-week period (**Figure 1H and 1I**). Gel electrophoresis confirmed the persistence of intact ASO bands without apparent degradation smear during incubation (**Figure S2A**). AuNP showed no marked increase in hydrodynamic diameter (**Figure 1J and S2B**), and its PDI and UV–vis absorbance profile remained largely unchanged over four weeks, indicating no evident aggregation or colloidal destabilization under the tested conditions (**Figure S2C and S2D**). Together, these stability data support the suitability of the model-agent cocktail for four-week infusion.

This design allowed direct comparison between sustained infusion into the diploic space and infusion beyond the inner skull cortex while confirming that the model agents retained analytical integrity or colloidal stability during the infusion-relevant period.

### Anatomical validation of nanoIPI placement by CT and micro-CT scans

**Figure 2:**
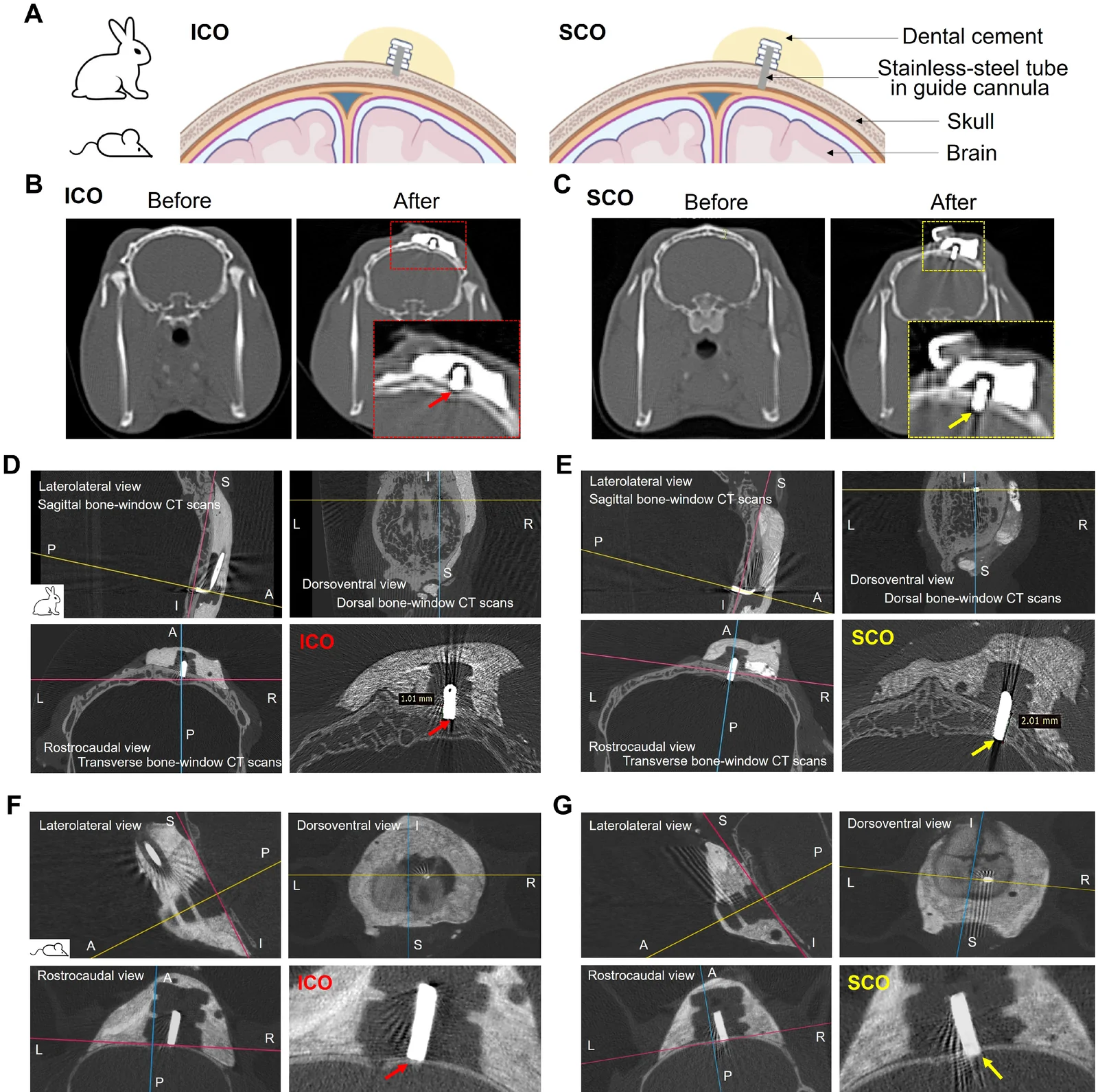
** CT and micro-CT validation of ICO and SCO placement in mice and rabbits (A)** Schematic illustration of ICO and SCO cannula placement in mice and rabbits. In ICO, the cannula terminus is positioned within the skull diploic space; in SCO, the terminus is positioned beyond the inner skull cortex. **(B, C)** Conventional CT images obtained before and after surgery in live rabbits under anesthesia. Close-up images show the ICO cannula terminus within the diploic space (**B**, red arrow) and the SCO cannula terminus beneath the skull after passing beyond the inner cortex (**C**, yellow arrow). **(D, E)** Micro-CT-based 3D MPR images of dissected rabbit skulls after four weeks, showing lateral, rostrocaudal, and dorsoventral views of ICO **(D)** and SCO **(E)** placement. Corresponding 3D volume-rendered images are shown in Figure S5. Scale bars, 5.0 mm. Directional labels indicate right lateral (R), left lateral (L), anterior **(A)**, posterior (P), superior (S), and inferior **(I)**. **(F, G)** Micro-CT validation of ICO **(F)** and SCO **(G)** placement in live mice one week after surgery. Red arrows indicate ICO termini within the diploic space, and yellow arrows indicate SCO termini positioned beyond the inner skull cortex.

Before the surgical procedure to install ICO or SCO with the nanoIPI, we performed conventional CT scans to investigate the skull thickness of each rabbit, as shown in **Figure S3**. The average skull thickness at the targeted location was measured to be 2.19 ± 0.04 mm (**Table S1**). Surgery for skull drilling in the right parietal bone was carefully performed in consideration of the individual rabbits’ skull thickness. The detailed surgical procedure for ICO or SCO installation with the nanoIPI is presented in **Figure S4**. **Figure 2A** illustrates the installation of ICO and SCO in mice and rabbits. The diagram shows the placement of a stainless-steel tube within a guide cannula, terminating either into or beneath the skull for ICO and SCO, respectively. As shown in **Figure 2B and 2C**, we confirmed the anatomical placement of the nanoIPI in the ICO or SCO group through live CT scans one week post-operatively. In the ICO group, the terminus of the pump system was positioned within the diploic space of the skull bone (**Figure 2B,** red arrow). Contrarily, in the SCO group, the terminus was positioned beneath the skull bone, passing through the inner cortex (**Figure 2C,** yellow arrows). The position corresponds to the upper segment of the dural layer of the meninges.

To verify the accurate placement and stability of an installed nanoIPI for ICO and SCO, we conducted a micro-CT scan on the excised whole skull bone post-sacrifice. As shown in **Figure 2D and 2E**, 3D MPR images provided lateral, rostrocaudal, and dorsoventral views of the cannula placement, with corresponding 3D volume-rendered images shown in **Figure S5**. The images showed that the cannula terminus remained in the intended anatomical positions for four weeks. Furthermore, a transverse bone-window micro-CT scan image of the ICO clearly demonstrates that the terminus of the customized guide cannula was positioned at a depth of 1 mm between the inner and outer cortex of the skull bone (**Figure 2D and S6**). Conversely, the terminus of the customized guide cannula for SCO was situated beneath the skull, passing through the inner cortex, with a depth of 2 mm (**Figure 2E** and **S6**). These distinctions are also clearly visible in the 3D volume renderings, with red arrows indicating the termination point of the guide cannula for ICO and yellow arrows indicating the termination point of the guide cannula for SCO (**Figure S5**).

As shown in **Figure 2F and 2G**, we confirmed the successful installation of a nanoIPI in ICO or SCO in live mice through micro-CT scans. In the ICO group, the terminus of the stainless-steel tube was positioned inside the diploic space between the inner and outer cortex of the skull; in the SCO group, it was positioned beneath the skull bone, corresponding to the upper part of the dural layer of the meninges (as indicated by red arrow-ICO and yellow arrow-SCO). Quantitative micro-CT analysis confirmed that measured ICO cannula depths closely matched the intended target depths in both mice and rabbits, whereas SCO showed full-thickness skull penetration with dural exposure (**Table S2**). The inner skull cortex was preserved in all ICO animals and fully penetrated in all SCO animals, with a 100% placement success rate in each group. Together, CT and micro-CT confirmed that the species-adapted pump–cannula configuration reproducibly distinguished ICO from SCO in both species: ICO maintained the cannula terminus within the diploic space, whereas SCO positioned the terminus beyond the inner skull cortex. This anatomical distinction provided the basis for using SCO as a positive comparator for estimating ICO-mediated delivery when the inner skull cortex remains intact.

### Rabbit brain-associated exposure after sustained ICO

**Figure 3:**
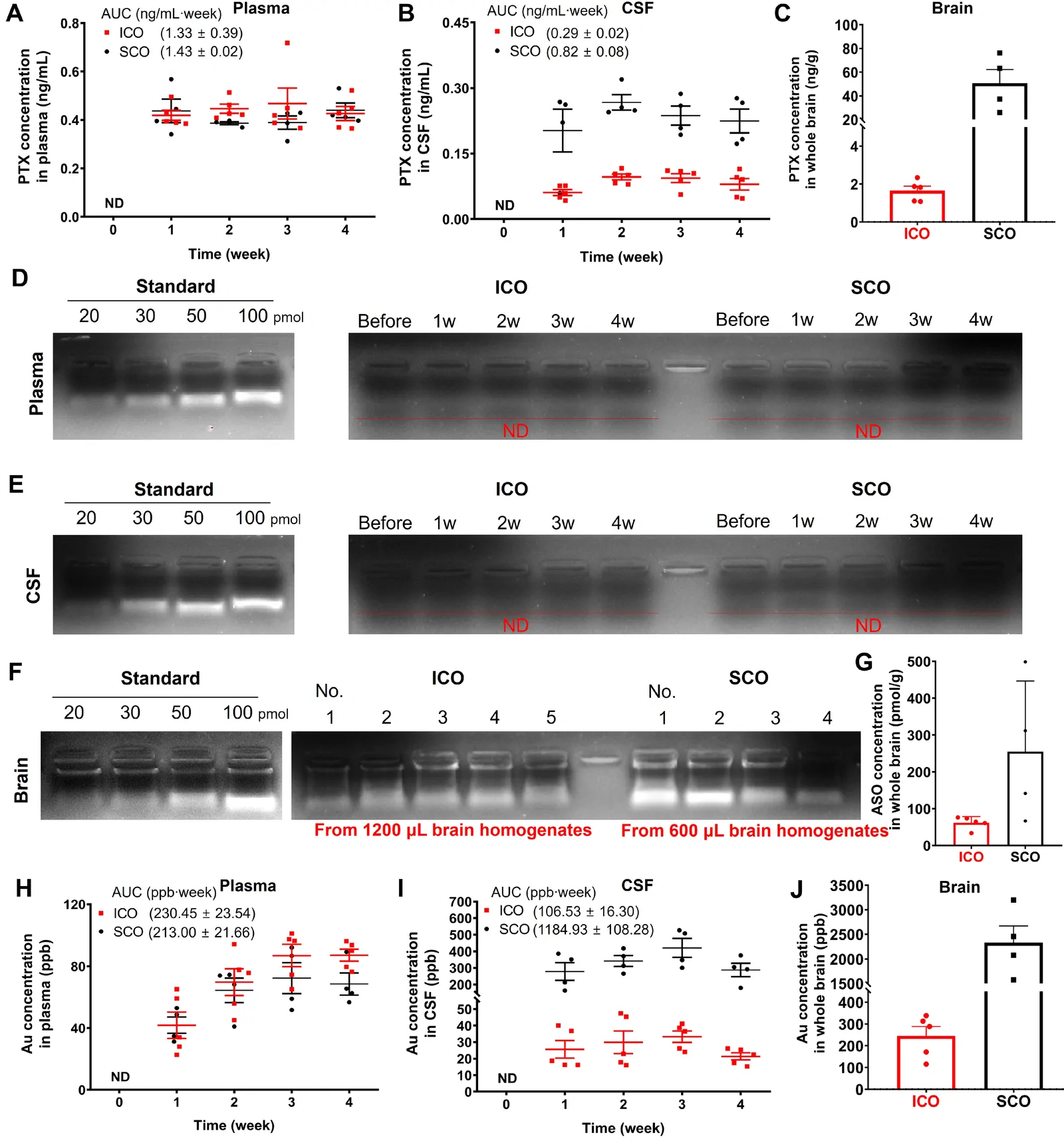
** Rabbit CSF and terminal brain-associated exposure after sustained ICO (n = 4-5). (A)** Plasma and **(B)** CSF concentration-time profiles of PTX over four weeks after ICO or SCO. AUC values are shown in parentheses next to each group label. **(C)** Terminal whole-brain PTX concentrations at week 4. **(D–F)** Representative gel images showing intact biotinylated ASO recovered from plasma **(D)**, CSF **(E)**, and brain homogenates **(F)**. Intact ASO was below the detection limit in plasma and CSF but was detected in terminal brain homogenates. **(G)** Quantification of terminal whole-brain ASO concentrations from gel band intensities. **(H, I)** Plasma **(H)** and CSF **(I)** concentration–time profiles of total gold over four weeks after ICO or SCO. **(J)** Terminal whole-brain total gold concentrations at week 4. Gold concentrations were quantified by ICP-MS. Data are presented as mean ± SEM; n = 4–5 per group.

In rabbits, brain-associated exposure after sustained ICO was evaluated using agent-specific quantitative assays for PTX, ASO, and AuNP, together with serial CSF sampling as a longitudinal CNS exposure index and terminal whole-brain concentrations at week 4 (**Figure 3**). Because brain exposure was quantified from bulk brain homogenates after removal of visible meningeal tissues, the measured concentrations were interpreted as brain-associated exposure rather than definitive parenchymal delivery.

PTX and AuNP were quantified by LC-MS/MS and ICP-MS, respectively, whereas intact biotinylated ASO was quantified using a streptavidin bead-based recovery and gel densitometry assay. To support semi-quantitative ASO analysis, matrix-matched calibration and extraction-recovery experiments were performed, showing linear densitometric responses over the detectable range and reproducible 50 pmol QC recovery in relevant tissue matrices (**Figure S7 and S8; Table S3**).

Because serial terminal brain collection was not feasible in rabbits, CSF AUC and terminal brain concentrations were interpreted together as complementary indicators of rabbit brain-associated exposure. For PTX and AuNP, plasma exposure was comparable between ICO and SCO, suggesting that differences in brain-associated exposure were not primarily driven by systemic exposure (**Figure 3A and 3H**). In contrast, CSF exposure was lower after ICO than after SCO, consistent with the presence of an inner skull cortex in the ICO condition (**Figure 3B and 3I**). Whole-brain PTX and AuNP concentrations at four weeks are shown in **Figure 3C and 3J**. ASO was not detected in plasma or CSF in either group (**Figure 3D and 3E**), likely due to concentrations below the detection limit. In terminal brain homogenates, however, intact ASO was detected in all rabbits at week 4 (**Figure 3F**), and quantitative analysis showed that ICO reached approximately 24% of the SCO brain level (**Figure 3G**). The pharmacokinetic parameters in rabbits after ICO or SCO were summarized in **Table 1**.

Overall, ICO in rabbits resulted in relative brain availability (F_CSF_) of 35.37% and 8.99% for PTX and AuNP, respectively. The terminal ICO/SCO brain concentration ratio (K_brain, 4 weeks_) was 3.26% for PTX, 24.22% for ASO, and 10.55% for AuNP. F_CSF_ and K_brain_ were interpreted as descriptive ratio-based indicators of ICO exposure relative to SCO exposure, with the corresponding AUC_CSF_ and terminal C_brain_ values reported as mean ± SEM in **Table 1**. Together, the rabbit data show that sustained ICO produced measurable brain-associated exposure of small-molecule, oligonucleotide, and nanoparticle agents, with ASO showing the highest terminal relative exposure.

### Mouse brain-associated exposure and regional distribution after sustained ICO

**Figure 4:**
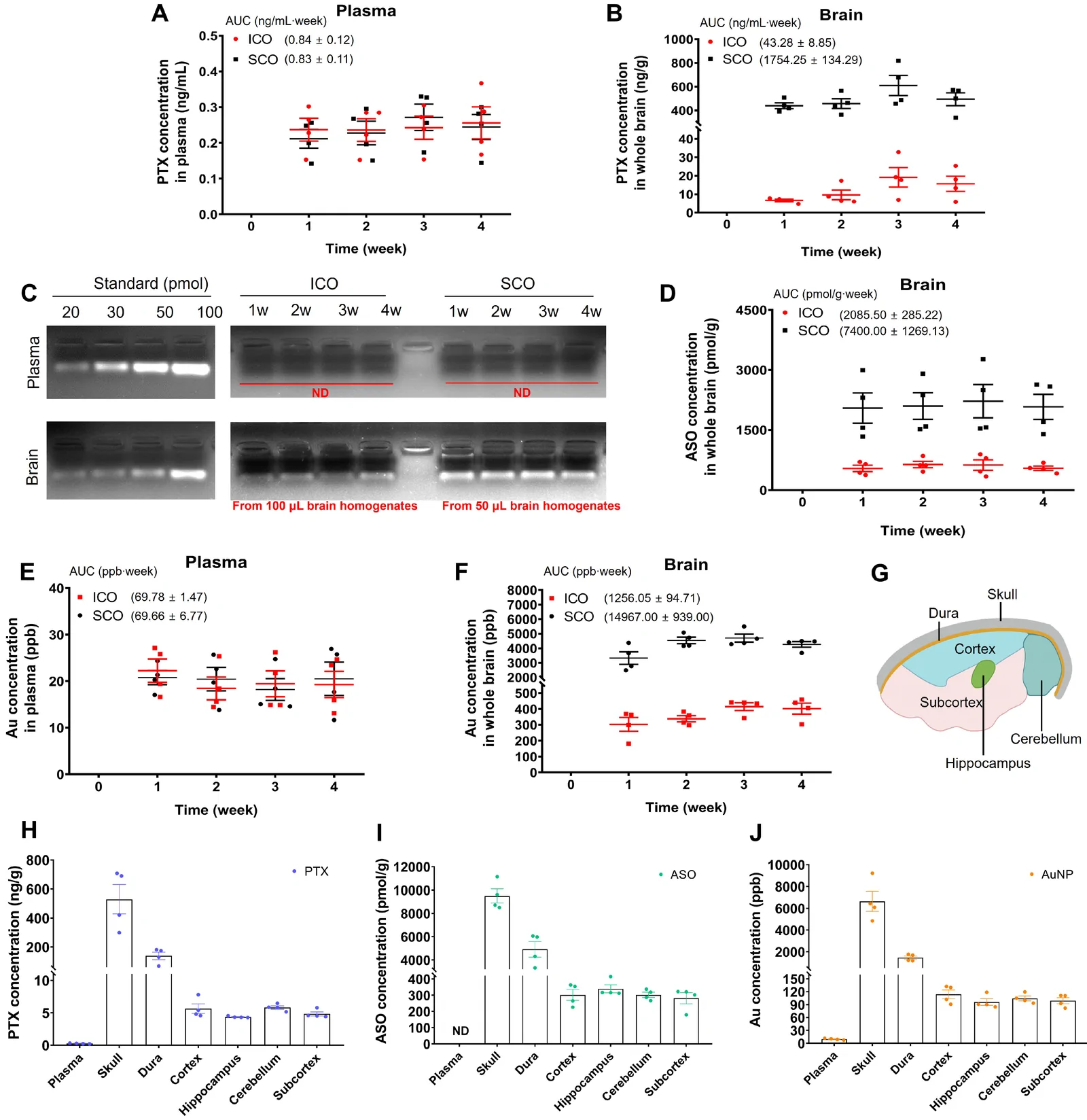
** Mouse brain-associated exposure and regional distribution after sustained ICO (n = 4). (A, B)** Plasma **(A)** and whole-brain **(B)** concentration–time profiles of PTX over four weeks after ICO or SCO. AUC values are shown in parentheses next to each group label. **(C)** Representative gel images showing intact biotinylated ASO recovered from plasma and brain homogenates. Intact ASO was below the detection limit in plasma but detected in brain homogenates. **(D)** Whole-brain ASO concentration–time profiles quantified from gel band intensities. **(E, F)** Plasma **(E)** and whole-brain **(F)** concentration–time profiles of total gold over four weeks after ICO or SCO. **(G)** Schematic of tissue sampling for regional biodistribution analysis, including skull, dura, cortex, hippocampus, cerebellum, and subcortex. **(H–J)** Biodistribution of PTX **(H)**, ASO **(I)**, and total gold/AuNP-associated signal **(J)** in plasma, skull, dura, and selected brain regions at week 4 after ICO. Data are presented as mean ± SEM; n = 4 per group.

Parallel mouse experiments supported the rabbit findings by showing measurable brain-associated exposure of all three model agents after four-week ICO (**Figure 4**). The pharmacokinetic parameters were summarized in **Table 2**. For PTX and AuNP, plasma exposure was comparable between ICO and SCO (**Figure 4A and 4E**), whereas brain-associated exposure was consistently lower after ICO (**Figure 4B and 4F**), reflecting the barrier imposed by the inner skull cortex. Intact ASO was detected in brain homogenates, not in plasma, over the four-week period (**Figure 4C and S9**), with quantitative profiles shown in **Figure 4D**. Calculated relative brain availabilities (F_brain_) were 2.47% (PTX), 28.18% (ASO), and 8.55% (AuNP). Terminal ICO/SCO brain concentration ratios were at week 4 were 3.17% for PTX, 26.38% for ASO, and 9.43% for AuNP. Although the exposure metrics differed between species, the terminal ICO/SCO brain concentration ratios showed a similar rank order, with ASO exhibiting the highest relative exposure. These findings support measurable skull-to-brain passage of a small molecule, an ASO surrogate, and nanoparticles after four-week ICO infusion.

To further characterize brain distribution after ICO, biodistribution analysis was performed across the skull, dura, cortex, hippocampus, cerebellum, and subcortex at four-week post-ICO (**Figure 4G – J; Table S4**). The gel band images of the intact form of ASO in these tissues are presented in **Figure S10**. As expected, the highest accumulation of all three agents was observed in the skull, followed by the dura. The relatively comparable concentrations across dissected brain regions are consistent with detectable exposure beyond the immediate infusion site, although the bulk-tissue design does not establish microscopic parenchymal distribution or exclude contributions from brain-associated meningeal/perivascular compartments. These mouse data supported cargo-dependent but reproducible brain-associated exposure after sustained ICO.

### Diploic-space localization and skull-associated passage of AuNP after ICO

**Figure 5:**
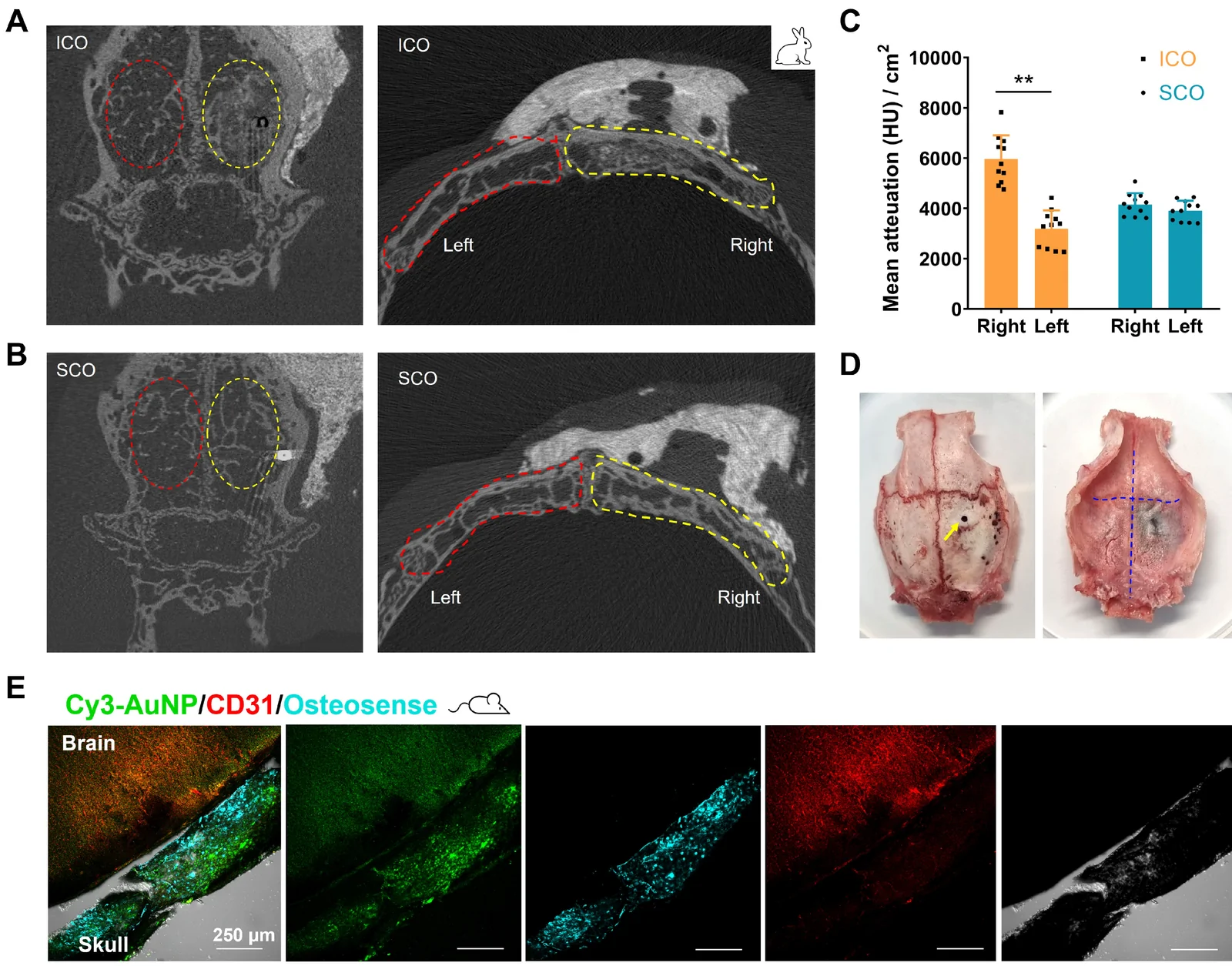
** Diploic-space localization and skull-associated passage of AuNP after ICO. (A, B)** Micro-CT images of dissected rabbit skulls four weeks after ICO **(A)** or SCO **(B)**, shown in dorsal and coronal planes. Yellow dotted lines indicate the right parietal region where the pump–cannula configuration was installed, and red dotted lines indicate the untreated left parietal region. **(C)** Mean attenuation per area of AuNP in the right and left parietal bone, derived from micro-CT scans on the dorsal plane. These values represent the mean attenuation of the region of interest (ROI) from eleven dorsal planes, considering only the diploic space and excluding the inner and outer cortex. Data are presented as mean ± SEM. For the statistically significant comparison in **(C)**, the paired mean difference between right and left parietal bone attenuation values was 2765 HU/cm² (95% confidence intervals (CI), 2312 to 3219; **p < 0.01).** (D)** Photographs of dissected rabbit skulls four weeks after ICO. The yellow arrow indicates the ICO cannula insertion site, and the blue dotted line outlines cranial bone boundaries. Black AuNP-associated signal was predominantly visible in the right parietal bone, consistent with ICO-side diploic-space localization. **(E)** Ex vivo confocal imaging of mouse coronal skull–brain sections one week after ICO with Cy3-labeled AuNP. Cy3-AuNP signals were observed in the ICO-side diploic space and adjacent brain-associated regions. CD31 and OsteoSense signals indicate vascular endothelium and mineralized skull bone, respectively. Scale bar, 250 μm

To examine diploic-space localization of the nanoparticle component after ICO, we tracked AuNP signals in excised whole skulls by micro-CT four weeks after infusion. In ICO-installed rabbit skulls, CT contrast was observed predominantly within the diploic space of the right parietal bone, where nanoIPI had been installed (**Figure 5A**). Conversely, the left parietal bone exhibited only the spongy bone structure. SCO-installed rabbit skull showed no apparent AuNP-associated contrast within the diploic space (**Figure 5B**). To quantify AuNP intensity, we measured the mean attenuation per area of AuNP in both the right and left parietal bone using micro-CT scans on the dorsal plane (**Figure 5C**). Quantification of mean attenuation per area showed approximately two-fold higher signal in the right parietal bone than in the left parietal bone after ICO, whereas no apparent right–left difference was observed after SCO. The presence of AuNP was visually confirmed in real photographs of the dissected rabbit skull after ICO for four weeks (**Figure 5D and S11**). Real photographs of dissected skulls further supported predominant AuNP localization in the right parietal bone after ICO. These findings support sustained localization of the AuNP component within the ICO-side diploic space.

Additionally, *ex vivo* skull-brain section imaging on coronal views was then performed using Cy3-labeled AuNP one week after ICO (**Figure 5E**). Cy3-labeled AuNP signals were observed in the ICO-side diploic space (bone marrow cavity of right parietal bone) and brain-associated regions, but not in the contralateral parietal bone (**Figure S12**). Because this imaging was *ex vivo* and endpoint-based, it should be interpreted as anatomical support rather than real-time tracking of delivery kinetics. All the imaging data support sustained localization of the AuNP component within the ICO-side diploic space and suggest passage from the skull compartment toward brain-associated compartments.

### Systemic and neuroinflammatory tolerability after sustained ICO in mice and rabbits

**Figure 6:**
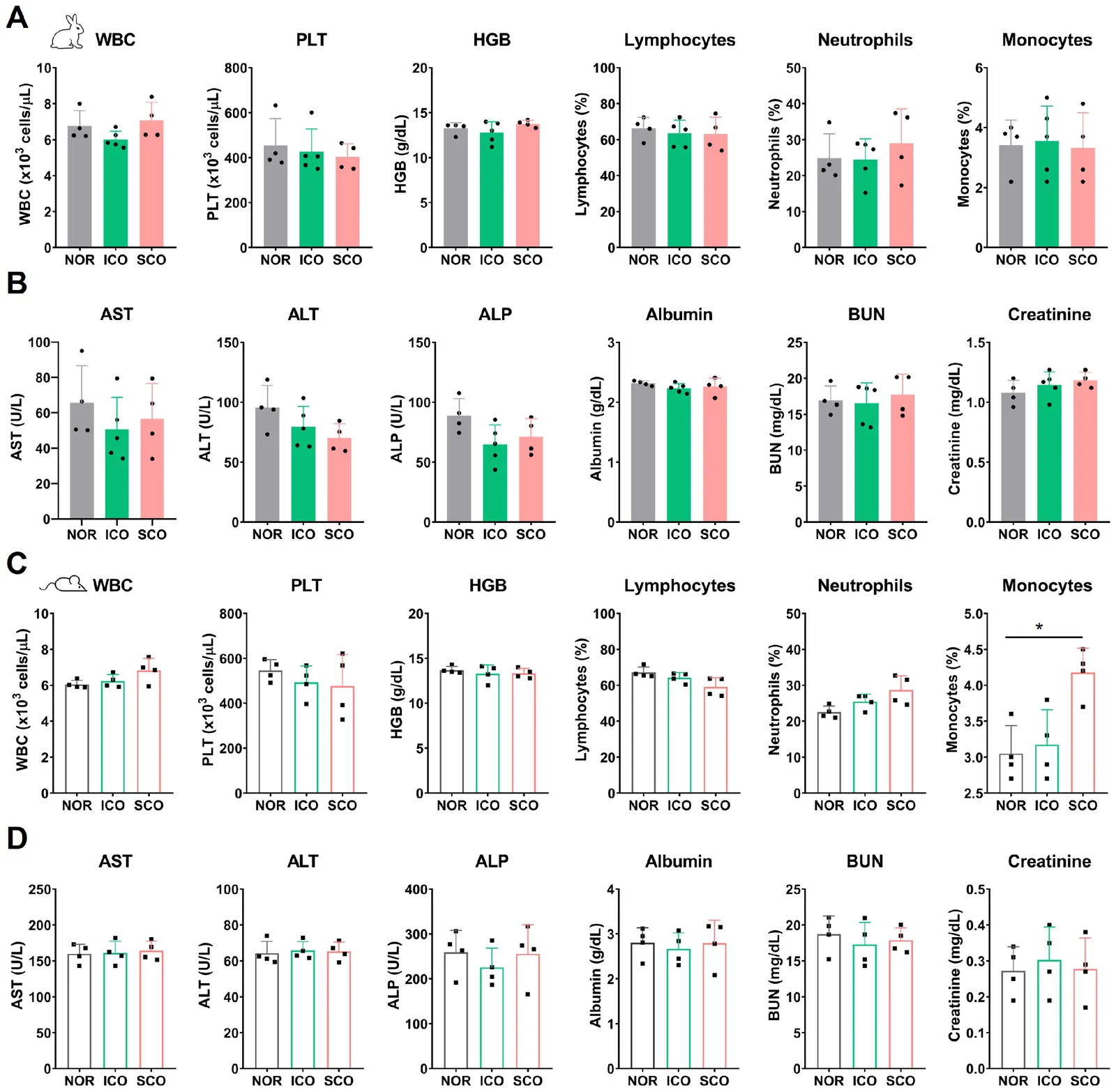
** Hematological markers and serum biochemical parameters in mice and rabbits after ICO or SCO. (A)** Complete blood cell count parameters in rabbits, including WBC (white blood cell count), PLT (platelets), HGB (hemoglobin), Lymphocytes, Neutrophils and Monocytes after ICO or SCO **(B)** Serum biochemical parameters in rabbits, including AST (Aspartate Aminotransferase), ALT (Alanine Aminotransferase), ALP (Alkaline Phosphatase), albumin, BUN (Blood Urea Nitrogen) and creatinine after ICO or SCO. **(C, D)** Complete blood count **(C)** and serum biochemical parameters **(D)** in mice four weeks after ICO or SCO. NOR indicates preoperative baseline values collected seven days before surgery. Data are presented as mean ± SEM. For the statistically significant monocyte comparison in **(C)**, the mean difference between NOR and SCO was -1.125% (95% CI, -2.168 to -0.08205; *p < 0.05).

To assess systemic tolerability and immune-related response after four-week ICO or SCO, we performed hematological and serum biochemical analyses in mice and rabbits. Most hematological parameters, including white blood cells, platelets, hemoglobin, lymphocytes, neutrophils, and monocytes, did not differ significantly from the normal group in either species (**Figure 6A and 6C; Tables S5 and S7**), except for increased monocytes in the mouse SCO group. Serum biochemical metrics, including AST, ALT, ALP, albumin, BUN, and creatinine, also exhibited no statistically significant differences between ICO/SCO-treated animals and normal controls under the current sample size and detection conditions (**Figure 6B and 6D; Tables S6 and S8**). Body weight was not significantly changed after ICO or SCO in rabbits (**Table S9**) or mice (**Table S10**).

**Figure 7:**
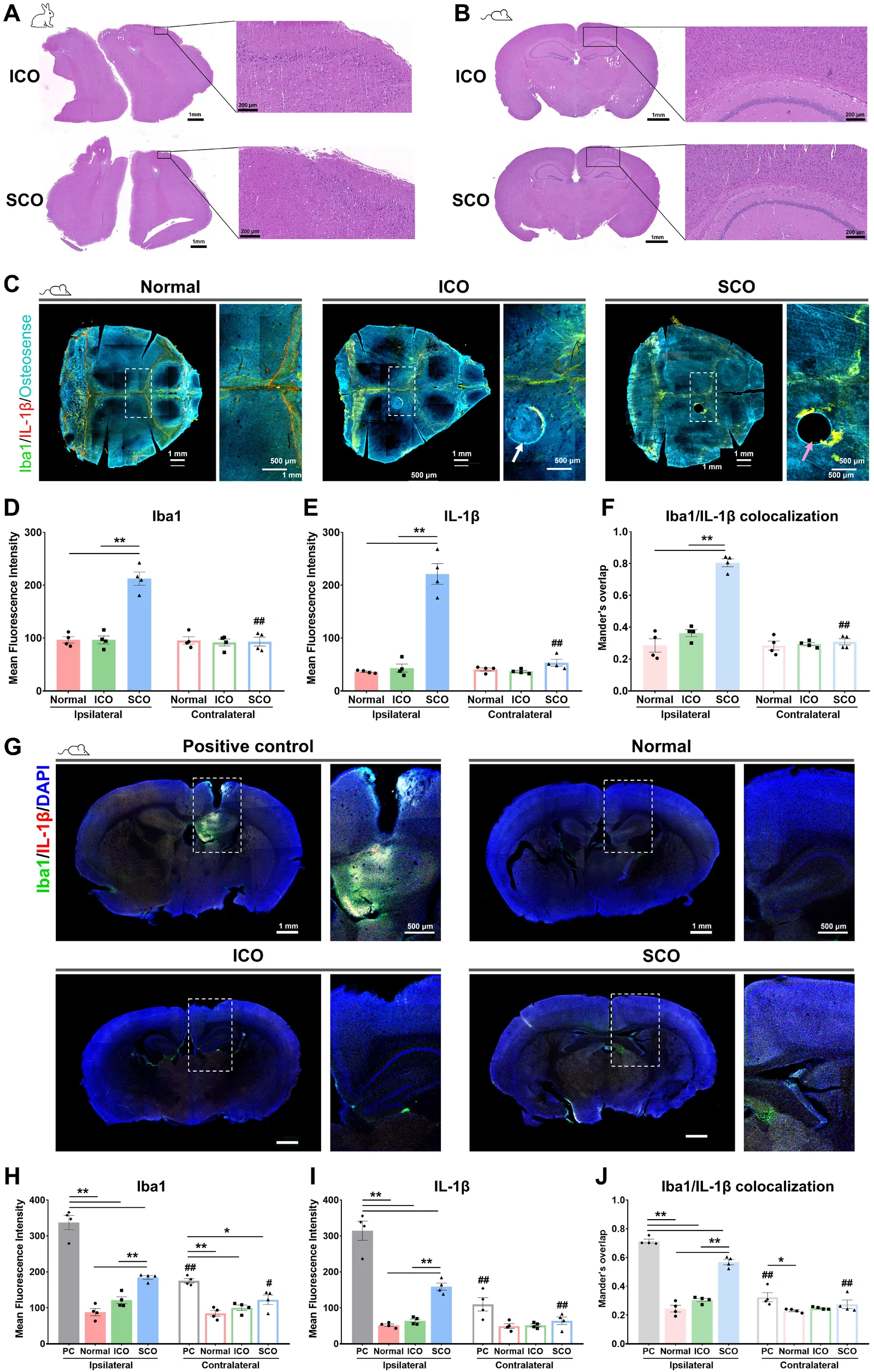
** Histopathological and neuroinflammatory assessment after ICO or SCO. (A, B)** Representative H&E-stained brain sections from rabbits **(A)** and mice **(B)** four weeks after ICO or SCO, showing no overt pathological abnormalities or tissue damage in the cerebral cortex. **(C)** Representative maximum-intensity projections of decalcified and cleared mouse skull whole-mounts four weeks after ICO or SCO. Samples were immunostained for Iba1 and IL-1β. Increased Iba1 and IL-1β fluorescence was observed at the SCO burr-hole site (pink arrow), whereas comparable signals were not apparent at the ICO thinned-skull site (white arrow). Quantification of Iba1 **(D)** and IL-1β **(E)** mean fluorescence intensity in the ipsilateral skull region containing the drilling site (right parietal bone) and the contralateral non-drilled skull region (left parietal bone). **(F)** Manders’ overlap coefficient for Iba1/IL-1β colocalization in skull.** (G)** Representative mouse brain sections imaged at 4× magnification four weeks after ICO or SCO. An ICV-injected mouse was used as a positive control for neuroinflammatory responses, and a healthy mouse served as the normal control. Quantification of Iba1 **(H)** and IL-1β **(I)** mean fluorescence intensity in ipsilateral and contralateral brain regions. **(J)** Manders’ overlap coefficient for Iba1/IL-1β colocalization in brain sections. Data are presented as mean ± SEM (n = 4). Statistical significance within ipsilateral or contralateral regions among positive control, normal, ICO, and SCO groups is indicated by *p < 0.05 and **p < 0.01. Statistical significance between ipsilateral and contralateral regions within the same treatment group is indicated by #p < 0.05 and ##p < 0.01.

Hematoxylin and eosin (H&E) staining revealed no overt pathological abnormalities or tissue damage in brain tissues from either species after ICO or SCO (**Figure 7A and 7B**). Representative maximum-intensity projections of decalcified/cleared mouse skulls and corresponding whole-brain immunofluorescence sections obtained four weeks post-treatment are shown in **Figure 7C and 7G**. Increased Iba1 and IL-1β signals were observed at the SCO burr-hole site (pink arrow), whereas comparable signals were not apparent at the ICO site (white arrow) (**Figure 7C and S13**). Consistently, brain immunostaining showed increased Iba1/CD68-associated microglial/macrophage activation markers 36, 37 and pro-inflammatory cytokine IL-1β signals 38-40 in cortical and subcortical regions after SCO, but not after ICO (**Figure 7G and S14**). To quantitatively assess neuroinflammatory responses, mean fluorescence intensities of Iba1, CD68, and IL-1β were measured using predefined regions of interest (ROIs), with four ROIs analyzed per animal. SCO showed increased Iba1-, CD68- and IL-1β-associated signals, whereas ICO did not show statistically significant increases in these markers under the present sample size and detection conditions (**Figure 7D, 7E, 7H and 7I; Figure S13B and S13C; Figure S14B and S14C**). In addition, Manders’ co-localization analysis of Iba1/IL-1β and Iba1/CD68 signals supported stronger myeloid/microglia-associated inflammatory activation after SCO than after ICO (**Figure 7F and 7J; Figure S13D and S14D**). Effect sizes and 95% confidence intervals are summarized in **Table S11 and S12**. Together, these results indicate that four-week ICO was not associated with overt systemic toxicity, histopathological abnormalities, or statistically significant increases in the measured neuroinflammatory markers under the current sample size and detection conditions. In contrast, SCO induced stronger local inflammatory signals, likely reflecting its more invasive penetration beyond the inner skull cortex.

## Discussion

This study supports the technical feasibility of sustained ICO infusion as a skull-to-brain-associated compartment delivery approach for prolonged CNS exposure of BBB-impermeable agents. Compared with our previous short-term ICO proof-of-concept study 18, the present work advances the field in four ways: first, by implementing a four-week nano-flow infusion paradigm; second, by scaling the procedure from mice to rabbits; third, by evaluating agents spanning a small molecule, an oligonucleotide surrogate, and a nanoparticle; and fourth, by using SCO as a positive comparator to estimate relative passage across the inner skull cortex. These data support sustained ICO infusion as a skull-based, extracerebral route for brain-associated drug exposure. However, several key limitations must be addressed before therapeutic application, including the absence of systemic control, incomplete understanding of the anatomical barriers, and the lack of disease-relevant efficacy data.

Recent experimental and review studies have increasingly recognized ICO as an alternative approach for brain drug delivery 2, 29, 41-47, reflecting growing interest in skull-based access routes that may support CNS-associated exposure without direct penetration into the brain parenchyma or CSF space. In this emerging context, the present study adds a distinct platform-level advance by moving ICO from short-term administration toward sustained infusion, cross-species implementation, and delivery of molecular and nanoscale agents.

The major conceptual advance in the present study is the transition from acute ICO administration to sustained ICO infusion. Chronic CNS diseases generally require prolonged drug exposure 5, and a single-dose delivery experiment cannot determine whether the skull diploic space can serve as a stable depot for long-term skull-to-brain transport. The four-week infusion design allowed us to assess time-dependent brain exposure, apparent approach to steady-state levels, terminal brain accumulation, and tolerability under prolonged implantation conditions.

The use of both mice and rabbits was important for evaluating the feasibility of ICO across different skull anatomies. Mice are useful for screening and mechanistic studies, but their thin calvarial bone and limited diploic space restrict the translational interpretation of ICO procedures. Rabbits do not fully reproduce human cranial anatomy; nevertheless, their thicker parietal bone, larger diploic compartment, and more permissive surgical working space provide an intermediate scaling model for testing whether sustained ICO can be implemented beyond the rodent skull. In this study, species-adapted pump–cannula configurations enabled stable placement of the infusion terminus within the diploic space over four weeks, supporting the technical feasibility of sustained ICO infusion in both species.

A key design feature of this study was the use of SCO as a positive comparator rather than systemic IV administration. IV administration is useful for comparing ICO with BBB-limited systemic delivery, but it does not directly estimate the contribution of the skull inner cortex to ICO-mediated transport. SCO positions the cannula terminus beyond the inner skull cortex and therefore provides a practical upper-bound comparator for the exposure achievable when this anatomical barrier is circumvented. However, SCO should not be interpreted as an absolute 100% brain-availability reference. The ICO/SCO ratios reported here are relative exposure indicators, not absolute bioavailability values.

The absence of an IV control group limits direct assessment of the relative advantage of ICO over systemic administration. Prior studies have reported that paclitaxel, oligonucleotides, and gold nanoparticles generally show restricted brain exposure after systemic administration because of BBB efflux 33, molecular size and charge 48, or limited nanoparticle penetration 49. Although these literature values could provide useful information on brain exposure after systemic administration of the agents used in this study, differences in dosing regimen, species, analytical methods, and exposure duration limit direct comparison with the present sustained ICO paradigm. Nevertheless, these reports help contextualize the present findings by indicating that the measurable brain-associated exposure achieved after sustained ICO infusion is notable for agents that are typically restricted by systemic BBB-limited delivery. Future studies incorporating matched IV or clinically relevant systemic comparator arms will be required to determine the relative advantage of sustained ICO infusion.

The relatively low ICO/SCO exposure ratios should be interpreted in the context of multiple anatomical barriers along the skull-to-brain interface. Although the key procedural difference between ICO and SCO is whether the inner skull cortex is preserved or circumvented, transport from the skull or subcalvariosseous compartment toward brain-associated tissues may still be constrained by the dura, arachnoid, perivascular interfaces, and other meningeal barriers. Therefore, the ICO/SCO ratios likely reflect not only permeability across the inner skull cortex, but also the rate-limiting effects of downstream meningeal interfaces, cargo-specific tissue binding, retention, and clearance. Moreover, BBB integrity was not directly evaluated in the present study. Thus, the present results support measurable skull-to-brain-associated exposure after ICO, but do not demonstrate unrestricted parenchymal transport or direct physiological bypass of the BBB. Future studies incorporating BBB integrity assays and spatially resolved distribution analyses will be required to define the anatomical and physiological barriers governing ICO-mediated transport.

The relative ICO/SCO brain exposure differed among the three model agents. ASO showed the highest relative brain exposure, whereas PTX showed the lowest. This difference should not be interpreted solely as size-dependent permeability across the inner skull cortex. The observed ratios likely reflect a combination of skull-to-brain transport, tissue binding, local retention, clearance, and analytical recovery. In particular, phosphorothioate-modified ASOs can exhibit strong tissue association, whereas PTX and AuNP may be governed by distinct distribution and retention kinetics. Therefore, the present results support cargo-dependent skull-to-brain exposure after sustained ICO, but do not define a simple molecular-size cutoff for ICO-mediated delivery.

The exposure metrics also differed between species. In mice, independent cohorts allowed weekly terminal brain collection and direct estimation of brain AUC. In rabbits, serial CSF sampling was used to reduce animal use, and terminal brain concentrations were measured at week 4. Therefore, rabbit F_CSF_ and mouse F_brain_ should be interpreted as complementary but non-identical indicators of CNS exposure. CSF concentrations do not necessarily represent regional parenchymal concentrations, particularly for agents with strong tissue binding or limited CSF mobility. This distinction is important when comparing ICO performance across species and cargo types.

Analysis of bulk brain homogenates cannot clearly separate actual parenchymal exposure from signals originating in adjacent meningeal or perivascular compartments. Although visible meningeal tissues were removed before regional brain dissection, a small amount of microscopic residual meningeal tissues may have remained in the brain homogenates, because the skull, dura, and cortical surface are anatomically apposed along the ICO delivery route. For this reason, the brain homogenate results are more appropriately interpreted as brain-associated exposure rather than definitive evidence of delivery into the brain parenchyma.

Sustained ICO infusion may occupy a distinct translational niche between systemic BBB-limited delivery and direct CSF or parenchymal administration. CSF-based administration routes such as intracerebroventricular (ICV) and intrathecal (IT) delivery have enabled clinical translation of biologics and nucleic acid therapeutics for selected CNS disorders 10, 11, 50, but they require direct access to CSF spaces and are associated with procedure-related risks, including infection, hemorrhage, CSF leakage, and neural injury 51, 52. In this context, ICO may offer a distinct skull-based access route because the infusion terminus remains within the diploic space without intentional penetration into the CSF or brain parenchyma. While ICO still involves skull drilling and device implantation, its translational appeal is not that it eliminates invasiveness, but that it relocates sustained CNS access from the CSF or brain parenchyma to the skull diploic space.

The safety profile of sustained ICO infusion should be interpreted with caution. In this study, four-week ICO was not associated with overt histopathological abnormalities or statistically significant increases in the measured neuroinflammatory markers, whereas SCO induced stronger local inflammatory signals, likely reflecting its more invasive penetration beyond the inner skull cortex. However, the absence of statistically significant increases in Iba1, CD68, or IL-1β after ICO should not be interpreted as definitive evidence of no neuroinflammatory response. Because this exploratory feasibility study used a limited sample size, small-to-moderate inflammatory changes may have gone undetected. Larger longitudinal studies using additional inflammatory and glial activation markers will be required to further define the long-term neuroimmune safety profile of sustained ICO infusion.

Several limitations should be acknowledged. This study was designed to establish the feasibility of sustained ICO delivery rather than disease-specific therapeutic efficacy; therefore, disease models and pharmacodynamic endpoints were not included. The biotinylated scramble ASO should be interpreted as a delivery surrogate, not as a functional therapeutic ASO. Although AuNP imaging and regional biodistribution support skull-to-brain-associated passage, the precise microscopic route across the skull–meningeal–brain interface remains unresolved. Moreover, bulk-tissue analysis cannot define cell-type-specific uptake, intracellular localization, or pharmacologically active concentrations in target brain cells. Future studies should validate disease-relevant efficacy using functional agents and further examine long-term tolerability and microscopic transport mechanisms.

## Conclusion

The present study provides preclinical evidence supporting the technical feasibility of sustained ICO infusion in mice and rabbits for a wide range of therapeutic agents. By adapting a species-specific nano-flow pump–cannula configuration to skull anatomy, sustained ICO maintained diploic-space delivery over four weeks and produced measurable brain-associated exposure of BBB-impermeable agents spanning a small molecule, an oligonucleotide surrogate, and a nanoparticle. Although therapeutic efficacy, systemic comparator studies, microscopic transport mechanisms, and long-term safety remain to be established, these findings support sustained ICO infusion as a technically feasible extracerebral skull-compartment approach for controlled and prolonged exposure of CNS therapeutics limited by the BBB.

## Supplementary Material

Supplementary figures and tables.

## Figures and Tables

**Table 1:** Summary of pharmacokinetic parameters in rabbits after ICO or SCO. (n = 4-5, mean ± SEM)

Parameters	Groups					
	PTX		ASO		AuNP	
	ICO	SCO	ICO	SCO	ICO	SCO
AUC_plasma_ ng/mL ∙week (pmol/mL∙week)	1.33 ± 0.39 (1.56 ± 0.46)	1.43 ± 0.02 (1.67 ± 0.02)	ND	ND	230.45 ± 23.54	213.00 ± 21.66
AUC_CSF_ ng /mL∙week (pmol/mL∙week)	0.29 ± 0.02 (0.34 ± 0.02)	0.82 ± 0.08 (0.96 ± 0.09)	ND	ND	106.53 ± 16.30	1184.93 ± 108.28
C_brain, 4 weeks_ ng/g (pmol/g)	1.65 ± 0.24 (1.93 ± 0.28)	50.64 ± 11.55 (59.30 ± 13.53)	800.02 ± 98.20 (61.64 ± 7.57)	3303.71 ± 1247.43 (254.56 ± 96.12)	245.35 ± 43.26	2326.02 ± 342.85
F_CSF_	35.37		-		8.99	
K_brain, 4 weeks_	3.26		24.22		10.55	

**Table 2:** Summary of pharmacokinetic parameters in mice after ICO or SCO. (n = 4, mean ± SEM)

Parameters	Groups					
	PTX		ASO		AuNP	
	ICO	SCO	ICO	SCO	ICO	SCO
AUC_plasma_ ng/mL ∙week (pmol/mL∙week)	0.84 ± 0.12 (0.98 ± 0.14)	0.83 ± 0.11 (0.97 ± 0.13)	ND	ND	69.78 ± 1.47	69.66 ± 6.77
AUC_brain_ ng /mL∙week (pmol/mL∙week)	43.28 ± 8.85 (50.68 ± 10.36)	1754.25 ± 134.29 (2054.38 ± 157.27)	27066.31 ± 3701.65 (2085.50 ± 285.22)	96039.64 ± 16471.25 (7400.00 ± 1269.13)	1256.05 ± 94.71	14697.00 ± 939.00
C_brain, 4 weeks_ ng/g (pmol/g)	15.66 ± 4.10 (18.34 ± 4.80)	494.59 ± 54.30 (579.21 ± 63.58)	2882.55 ± 214.13 (222.11 ± 16.50)	7149.73 ± 784.26 (550.90 ± 60.43)	401.98 ± 34.43	4264.85 ± 195.27
F_brain_	2.47		28.18		8.55	
K_brain, 4 weeks_	3.17		26.38		9.43	

## Data Availability

The data used and/or analyzed during the current study are available from the corresponding author on reasonable request.

## References

[1] Terstappen GC, Meyer AH, Bell RD, Zhang W (2021). Strategies for delivering therapeutics across the blood-brain barrier. Nat Rev Drug Discov.

[2] Wang X, Yin Y, Zhou H, Chi B, Guan L, Li P (2025). Drug delivery pathways to the central nervous system via the brain glymphatic system circumventing the blood-brain barrier. Exploration.

[3] Upton DH, Ung C, George SM, Tsoli M, Kavallaris M, Ziegler DS (2022). Challenges and opportunities to penetrate the blood-brain barrier for brain cancer therapy. Theranostics.

[4] Wu D, Chen Q, Chen X, Han F, Chen Z, Wang Y (2023). The blood-brain barrier: structure, regulation, and drug delivery. Signal Transduct Target Ther.

[5] Nance E, Pun SH, Saigal R, Sellers DL (2022). Drug delivery to the central nervous system. Nat Rev Mater.

[6] Markowicz-Piasecka M, Darlak P, Markiewicz A, Sikora J, Kumar Adla S, Bagina S (2022). Current approaches to facilitate improved drug delivery to the central nervous system. Eur J Pharm Biopharm.

[7] Gong Z, Zhou D, Wu D, Han Y, Yu H, Shen H (2025). Challenges and material innovations in drug delivery to central nervous system tumors. Biomaterials.

[8] Wang J, Li Z, Pan M, Fiaz M, Hao Y, Yan Y (2022). Ultrasound-mediated blood-brain barrier opening: An effective drug delivery system for theranostics of brain diseases. Adv Drug Deliv Rev.

[9] Gorick CM, Breza VR, Nowak KM, Cheng VWT, Fisher DG, Debski AC (2022). Applications of focused ultrasound-mediated blood-brain barrier opening. Adv Drug Deliv Rev.

[10] Sadekar SS, Bowen M, Cai H, Jamalian S, Rafidi H, Shatz-Binder W (2022). Translational Approaches for Brain Delivery of Biologics via Cerebrospinal Fluid. Clin Pharmacol Ther.

[11] Fahoum F, Eyal S (2023). Intracerebroventricular administration for delivery of antiseizure therapeutics: Challenges and opportunities. Epilepsia.

[12] Mitusova K, Peltek OO, Karpov TE, Muslimov AR, Zyuzin MV, Timin AS (2022). Overcoming the blood-brain barrier for the therapy of malignant brain tumor: current status and prospects of drug delivery approaches. J Nanobiotechnology.

[13] Jeong SH, Jang JH, Lee YB (2023). Drug delivery to the brain via the nasal route of administration: exploration of key targets and major consideration factors. J Pharm Investig.

[14] Drath I, Richter F, Feja M (2025). Nose-to-brain drug delivery: from bench to bedside. Transl Neurodegener.

[15] Crowe TP, Greenlee MHW, Kanthasamy AG, Hsu WH (2018). Mechanism of intranasal drug delivery directly to the brain. Life Sci.

[16] Herisson F, Frodermann V, Courties G, Rohde D, Sun Y, Vandoorne K (2018). Direct vascular channels connect skull bone marrow and the brain surface enabling myeloid cell migration. Nat Neurosci.

[17] Kolabas ZI, Kuemmerle LB, Perneczky R, Forstera B, Ulukaya S, Ali M (2023). Distinct molecular profiles of skull bone marrow in health and neurological disorders. Cell.

[18] Kang JH, Ko YT (2023). Intraosseous administration into the skull: Potential blood-brain barrier bypassing route for brain drug delivery. Bioeng Transl Med.

[19] Yao H, Price TT, Cantelli G, Ngo B, Warner MJ, Olivere L (2018). Leukaemia hijacks a neural mechanism to invade the central nervous system. Nature.

[20] Cai R, Pan C, Ghasemigharagoz A, Todorov MI, Forstera B, Zhao S (2019). Panoptic imaging of transparent mice reveals whole-body neuronal projections and skull-meninges connections. Nat Neurosci.

[21] Brioschi S, Wang WL, Peng V, Wang M, Shchukina I, Greenberg ZJ (2021). Heterogeneity of meningeal B cells reveals a lymphopoietic niche at the CNS borders. Science.

[22] Cugurra A, Mamuladze T, Rustenhoven J, Dykstra T, Beroshvili G, Greenberg ZJ (2021). Skull and vertebral bone marrow are myeloid cell reservoirs for the meninges and CNS parenchyma. Science.

[23] Roth TL, Nayak D, Atanasijevic T, Koretsky AP, Latour LL, McGavern DB (2014). Transcranial amelioration of inflammation and cell death after brain injury. Nature.

[24] Mastorakos P, McGavern D (2019). The anatomy and immunology of vasculature in the central nervous system. Sci Immunol.

[25] Pulous FE, Cruz-Hernández JC, Yang C, Kaya Ζ, Paccalet A (2022). Cerebrospinal fluid can exit into the skull bone marrow and instruct cranial hematopoiesis in mice with bacterial meningitis. Nat Neurosci.

[26] Mazzitelli JA, Smyth LCD, Cross KA, Dykstra T, Sun J, Du S (2022). Cerebrospinal fluid regulates skull bone marrow niches via direct access through dural channels. Nat Neurosci.

[27] Estrada H, Razansky D (2022). Guided Waves in the Skull. In: Laugier P, Grimal Q, editors. Bone Quantitative Ultrasound: New Horizons. Cham: Springer International Publishing.

[28] Eisova S, Rangel de Lazaro G, Pisova H, Pereira-Pedro S, Bruner E (2016). Parietal Bone Thickness and Vascular Diameters in Adult Modern Humans: A Survey on Cranial Remains. Anat Rec.

[29] Kang JH, Yang JK, Cho KH, Lee OH, Kwon H, Kim SY (2024). Intracalvariosseous administration of donepezil microspheres protects against cognitive impairment by virtue of long-lasting brain exposure in mice. Theranostics.

[30] Aboghazleh R, Boyajian SD, Atiyat A, Udwan M, Al-Helalat M, Al-Rashaideh R (2024). Rodent brain extraction and dissection: A comprehensive approach. MethodsX.

[31] Kim S, Kang JH, Nguyen Cao TG, Kang SJ, Jeong K, Kang HC (2022). Extracellular vesicles with high dual drug loading for safe and efficient combination chemo-phototherapy. Biomater Sci.

[32] Howard-Lech VL, Lee TY, Craen RA, Gelb AW (1999). Cerebral blood volume measurements using dynamic contrast-enhanced x-ray computed tomography: application to isoflurane anaesthetic studies. Physiol Meas.

[33] Fellner S, Bauer B, Miller DS, Schaffrik M, Fankhanel M, Spruss T (2002). Transport of paclitaxel (Taxol) across the blood-brain barrier *in vitro* and *in vivo*. J Clin Invest.

[34] Xiong R, Ling G, Zhang Y, Guan J, Zhang P (2023). Nucleic acid delivery by ionizable nanocarriers for brain disease treatment. Brain-X.

[35] Etame AB, Diaz RJ, O'Reilly MA, Smith CA, Mainprize TG, Hynynen K (2012). Enhanced delivery of gold nanoparticles with therapeutic potential into the brain using MRI-guided focused ultrasound. Nanomedicine.

[36] Haynes SE, Hollopeter G, Yang G, Kurpius D, Dailey ME, Gan WB (2006). The P2Y12 receptor regulates microglial activation by extracellular nucleotides. Nat Neurosci.

[37] Hopperton KE, Mohammad D, Trepanier MO, Giuliano V, Bazinet RP (2018). Markers of microglia in post-mortem brain samples from patients with Alzheimer's disease: a systematic review. Mol Psychiatry.

[38] Kaneko N, Kurata M, Yamamoto T, Morikawa S, Masumoto J (2019). The role of interleukin-1 in general pathology. Inflamm Regen.

[39] Park SY, Kang MJ, Han JS (2018). Interleukin-1 beta promotes neuronal differentiation through the Wnt5a/RhoA/JNK pathway in cortical neural precursor cells. Mol Brain.

[40] Wang C, Wu Q, Zhuang L, Chen Y, Zhang Q, Wu Y (2025). Immunometabolism of macrophages in the bone microenvironment: a new perspective for bone healing therapy. J Adv Res.

[41] Song X, Qian H, Yu Y (2023). Nanoparticles Mediated the Diagnosis and Therapy of Glioblastoma: Bypass or Cross the Blood-Brain Barrier. Small.

[42] Zhang X, Liu L, Chai Y, Zhang J, Deng Q, Chen X (2024). Reimagining the meninges from a neuroimmune perspective: a boundary, but not peripheral. J Neuroinflammation.

[43] Eide Therkelsen H, Enger R, Eide PK, Ringstad G (2025). Evidence for cellular and solute passage between the brain and skull bone marrow across meninges: A systematic review. J Cereb Blood Flow Metab.

[44] Gao X, Liu X, Wang N, Cui C, Liu W, Yang M (2026). Nanoparticles hijack calvarial immune cells for CNS drug delivery and stroke therapy. Cell.

[45] Liu L, MacKinnon MJ, Atanasijevic T, Dodd S, Bouraoud N, Donahue D (2025). Direct delivery of MRI contrast through skull vessel/marrow pathways into the brain guided by microCT. Theranostics.

[46] Liu W, Yang M, Wang N, Liu X, Wang C, Shi K (2025). Intracalvariosseous injection: an approach for central nervous system drug delivery through skull bone marrow with a preclinical research in stroke. EBioMedicine.

[47] Wang N, Zhang M, Yang M, Liu W, Liu W, Ou Y (2025). Efficacy and safety of Y-3 intracalvariosseous injection versus intravenous injection in the treatment of acute large hemispheric infarction (SOLUTION-2): rationale and design of a multicentre, prospective, randomised, open-label, blind endpoint (PROBE) trial. Stroke Vasc Neurol.

[48] Geary RS, Norris D, Yu R, Bennett CF (2015). Pharmacokinetics, biodistribution and cell uptake of antisense oligonucleotides. Adv Drug Deliv Rev.

[49] Cheng Y, Dai Q, Morshed RA, Fan X, Wegscheid ML, Wainwright DA (2014). Blood-brain barrier permeable gold nanoparticles: an efficient delivery platform for enhanced malignant glioma therapy and imaging. Small.

[50] Roberts TC, Langer R, Wood MJA (2020). Advances in oligonucleotide drug delivery. Nat Rev Drug Discov.

[51] Atkinson AJ Jr (2017). Intracerebroventricular drug administration. Transl Clin Pharmacol.

[52] Power EA, Rechberger JS, Gupta S, Schwartz JD, Daniels DJ, Khatua S (2022). Drug delivery across the blood-brain barrier for the treatment of pediatric brain tumors - An update. Adv Drug Deliv Rev.

