# Supplementary material for: Nano-flow intracalvariosseous infusion enables skull-to-brain delivery of BBB-impermeable molecular and nanoscale agents in mice and rabbits
Source: Theranostics. 2026 Aug 24;16(15):8947–67. doi: 10.7150/thno.138110 (PMC13549374; doi:10.7150/thno.138110)
Supplement: Supplementary file 1 [file thnov16p8947s1.pdf]

Supplementary Materials

Nano-flow intracalvariosseous infusion enables skull-to-brain delivery of BBB-impermeable molecular and nanoscale agents in mice and rabbits

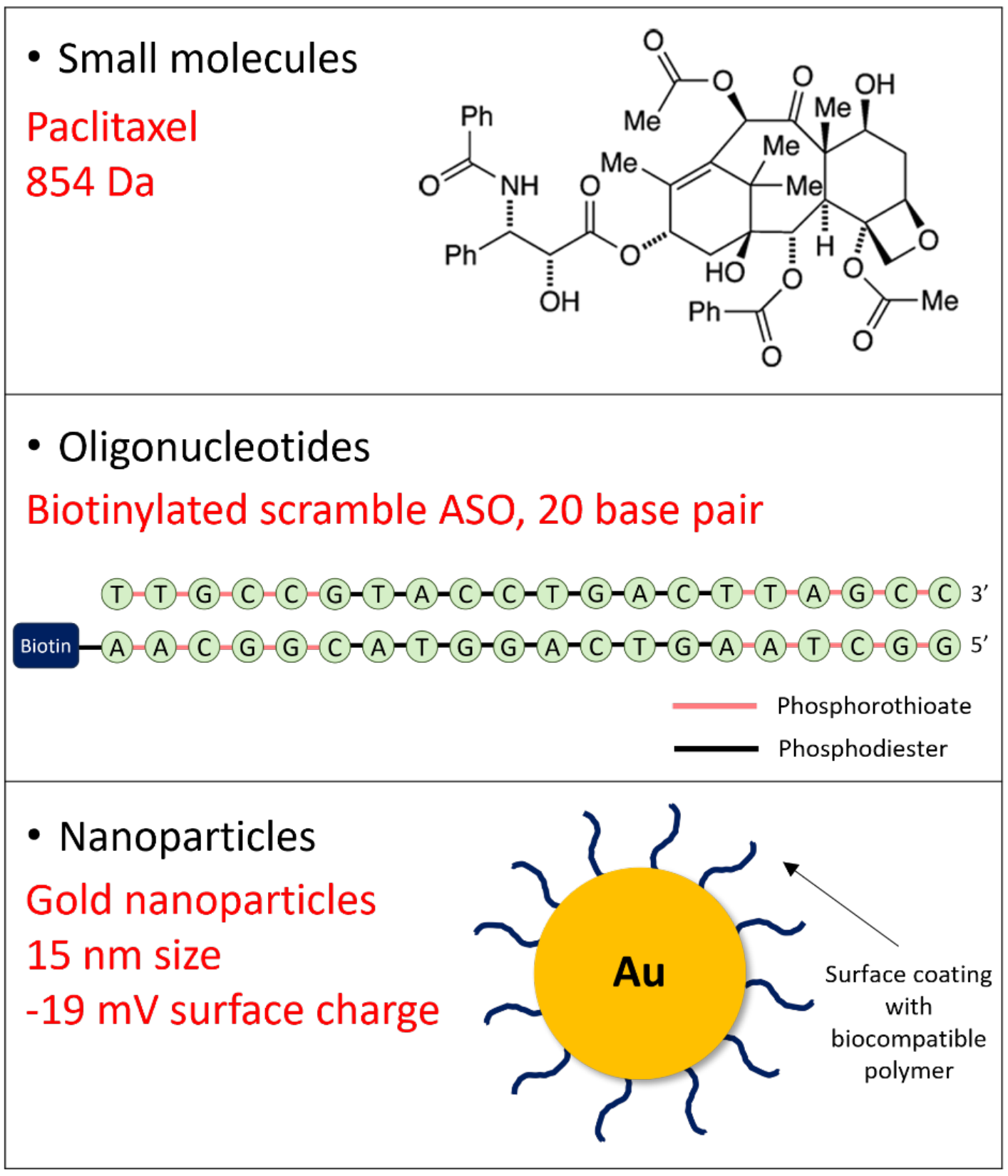

Figure S1. Physicochemical characteristics of the three model agents.

Paclitaxel (PTX; molecular weight, 854 Da), a 20-base pair phosphorothioate-modified biotinylated scramble antisense oligonucleotide (ASO), and 15-nm gold nanoparticles (AuNP) were selected as representative BBB-impermeable small-molecule, oligonucleotide, and nanoparticle agents, respectively. The ASO sequence and chemical modifications are shown. The three agents were formulated as a single cocktail for simultaneous infusion. No apparent interference was observed based on visual inspection and analytical recovery of each agent from the cocktail formulation.

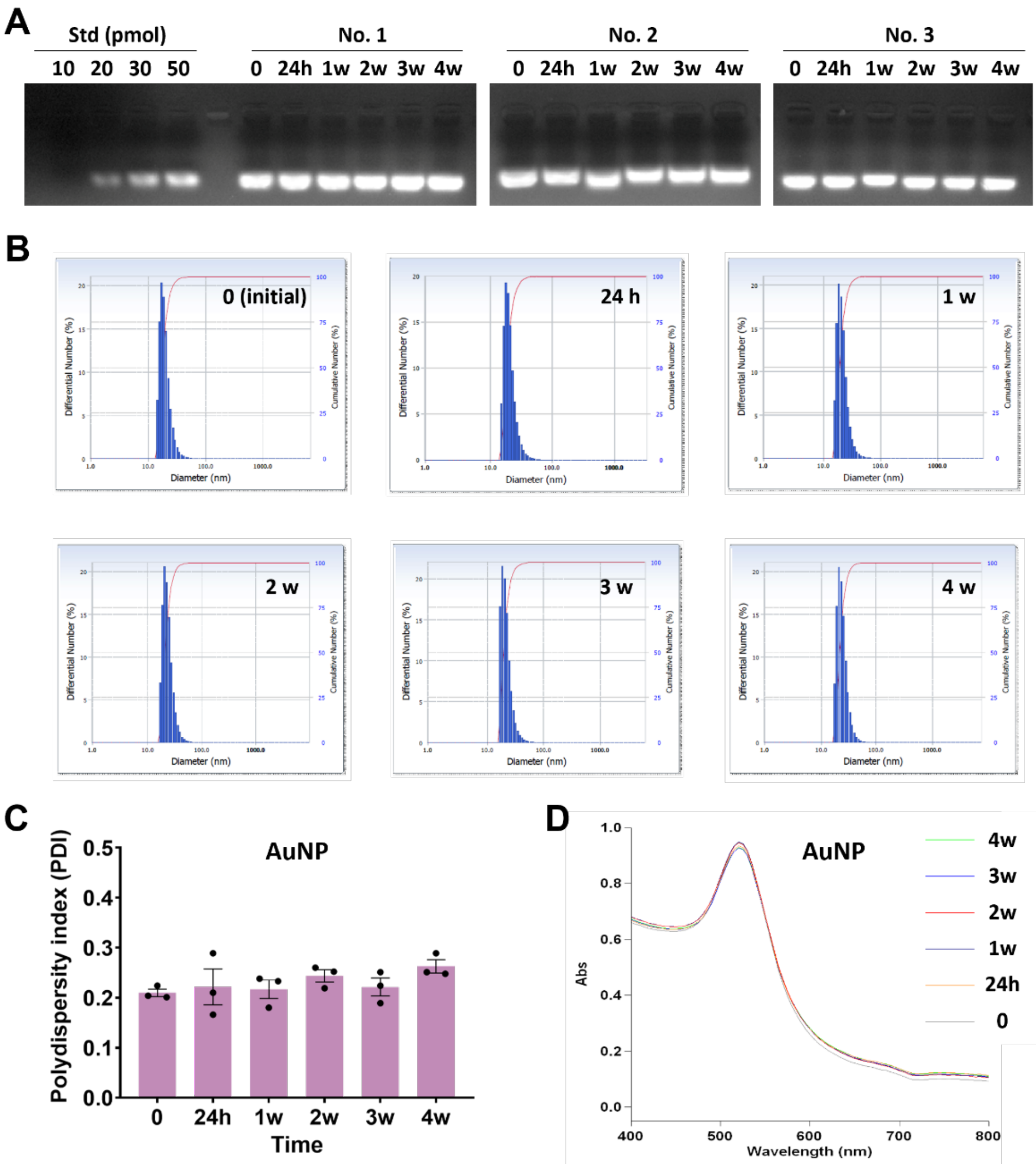

Figure S2. Stability assessment of intact ASO and AuNP under pump-relevant conditions. The model-agent cocktail was incubated at 37°C, and aliquots were collected at 0 h, 24 h, and 1, 2, 3, and 4 weeks. (A) Representative gel electrophoresis images showing intact biotinylated ASO bands without apparent degradation during incubation. (B) Representative DLS number-based size-distribution profiles of AuNP at each time point. (C) PDI values of AuNP measured by DLS. (D) Representative UV–vis absorbance spectra of AuNP. AuNP showed no marked changes in size distribution, PDI, or surface plasmon resonance profile over four weeks. Data are presented as mean ± SEM.

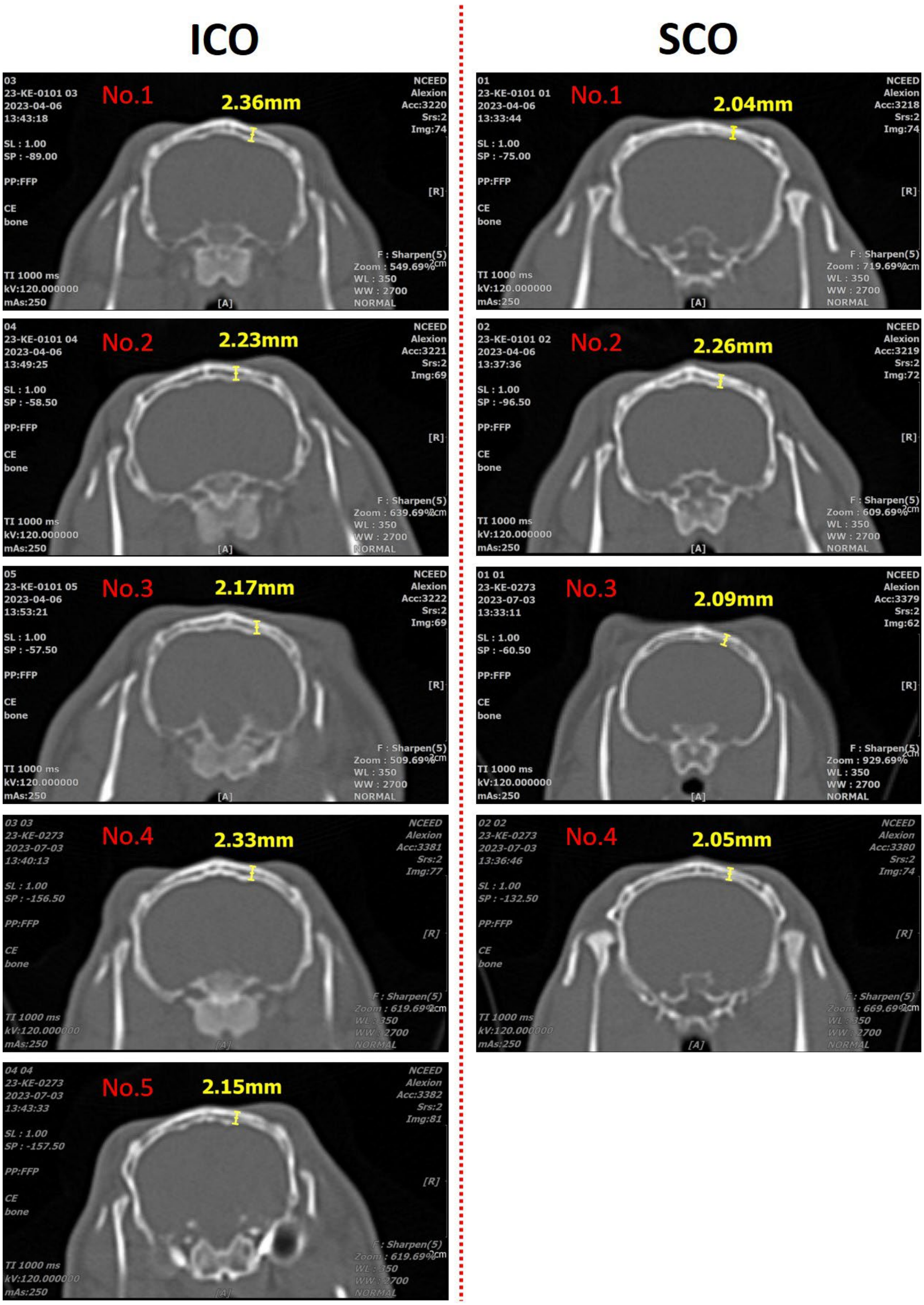

Figure S3. Preoperative CT-based measurement of rabbit skull thickness at the target drilling site.

Skull thickness at the planned cannula insertion site was measured in live rabbits before surgery using INFINITT CD Viewer software.

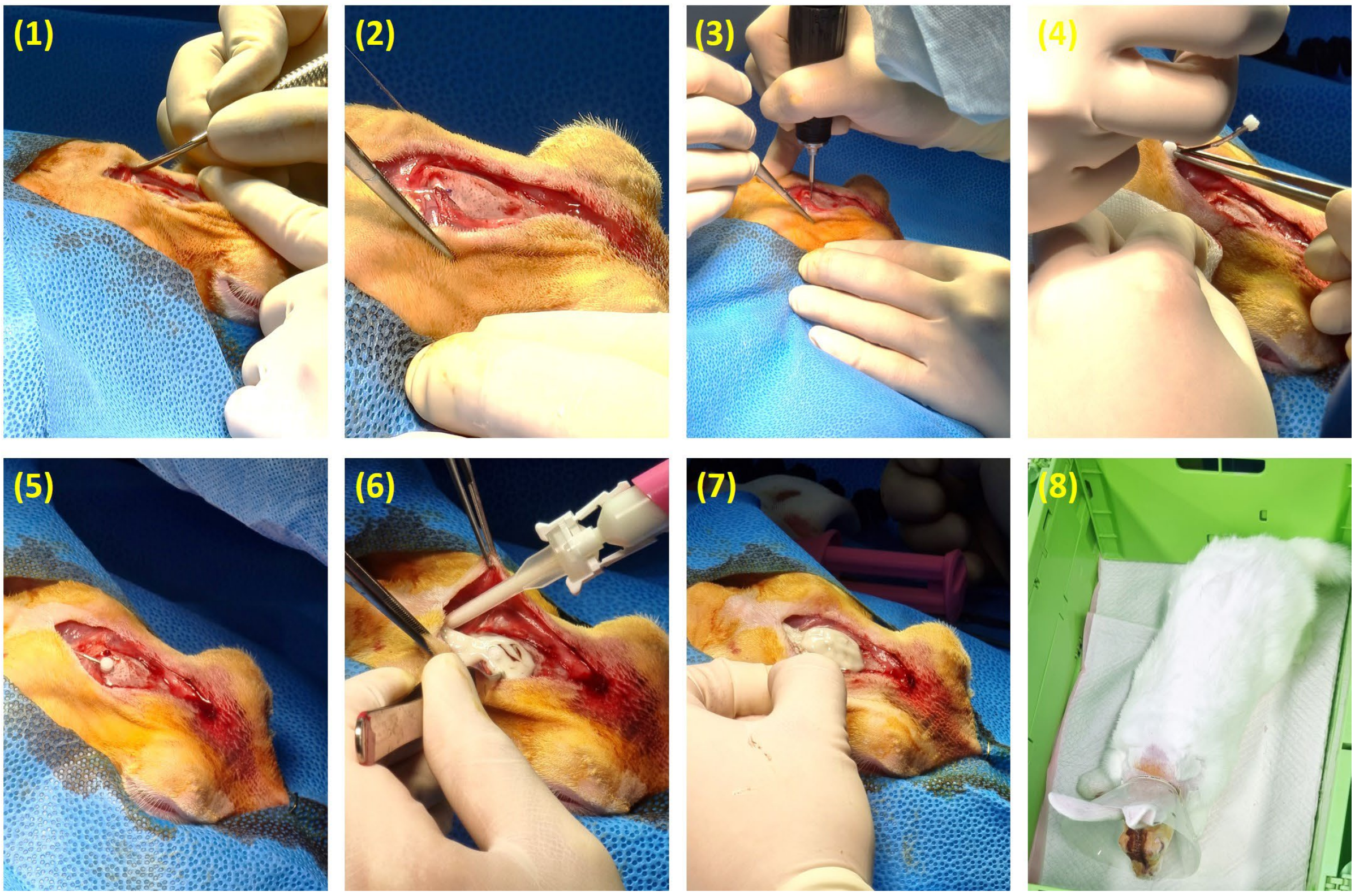

Figure S4. Surgical procedure for ICO or SCO pump–cannula implantation in rabbits.

The procedure was performed under anesthesia. (1) A midline skin incision was made from the middle of the skull toward the occipital protuberance. (2) The deep fascia and periosteum were dissected to expose the skull. (3) The right parietal bone was drilled at a position 5.0 mm lateral and 5.0 mm caudal to bregma to a depth of 1 mm for ICO or 2 mm for SCO. (4) The osmotic pump was placed in the right cervical subcutaneous space. (5) The guide cannula was inserted into the drilled skull site. (6) The cannula was secured using cyanoacrylate gel glue and resin-modified glass ionomer cement. (7) The wound was closed with sutures and surgical staples. (8) A neck collar was applied to protect the implanted pump–cannula system during the postoperative period.

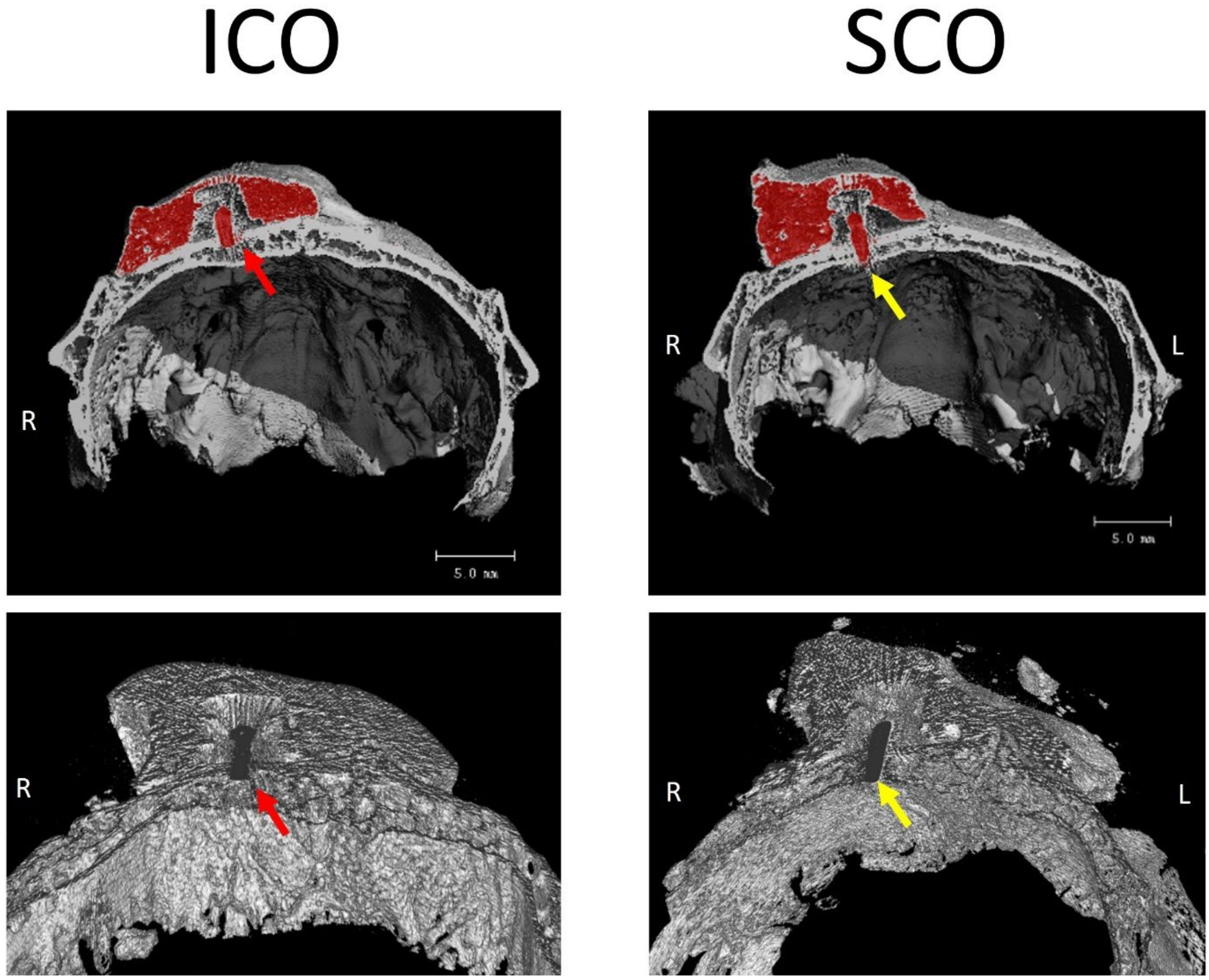

Figure S5. 3D volume-rendered CT and micro-CT validation of ICO and SCO placement in rabbits.

3D volume-rendered images of rabbit skulls showing ICO and SCO cannula placement. In the ICO group, the terminus of the customized guide cannula was positioned within the skull diploic space between the outer and inner skull cortices, as indicated by red arrows. In the SCO group, the cannula terminus was positioned beneath the skull after passing beyond the inner skull cortex, as indicated by yellow arrows. Dental cement and the stainless-steel guide cannula were pseudo-colored dark red for visualization. Scale bars, 5.0 mm. Directional labels indicate right lateral (R), left lateral (L), anterior (A), posterior (P), superior (S), and inferior (I).

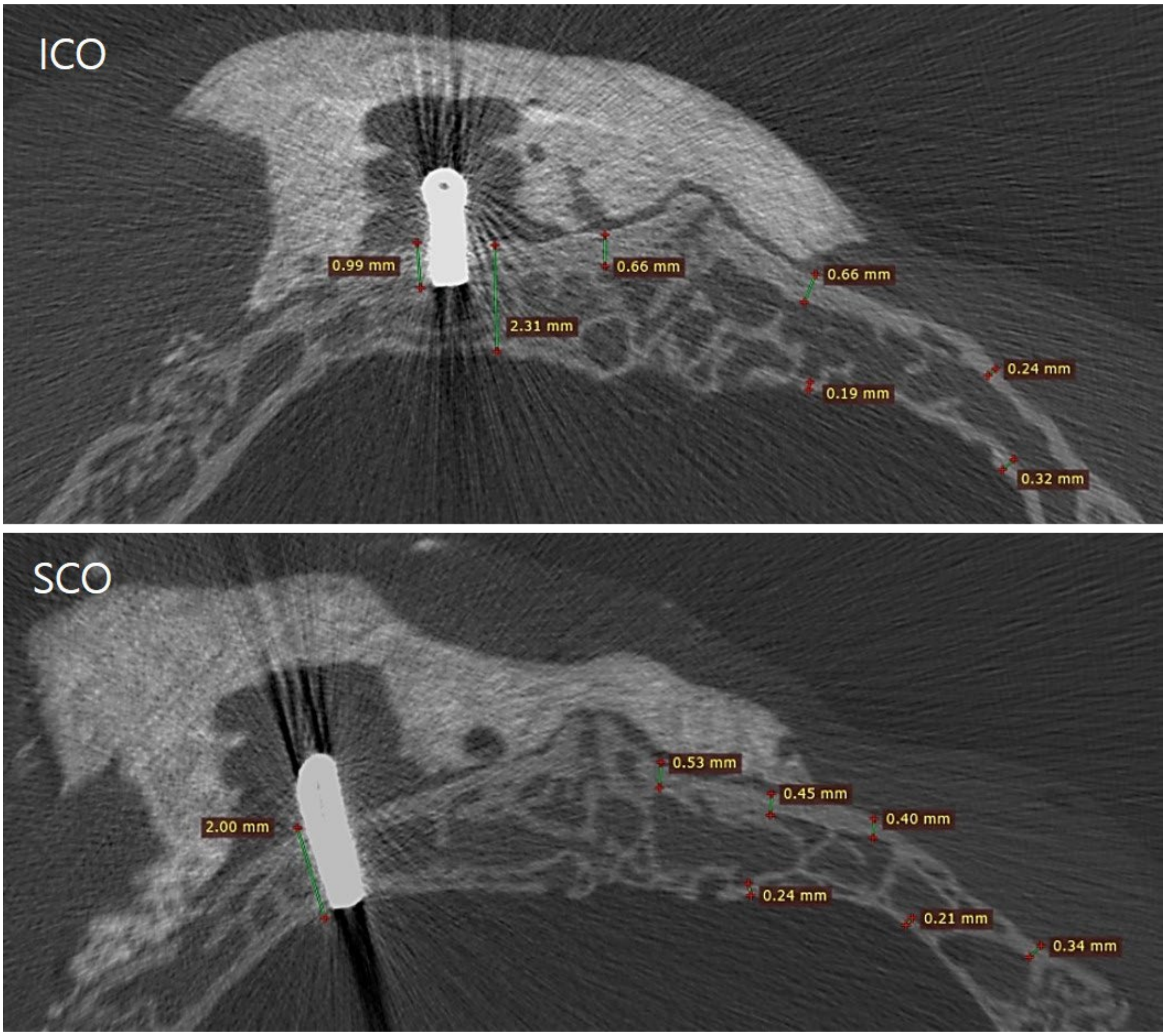

Figure S6. Magnified micro-CT images for measurement of cannula position and skull cortical thickness.

Magnified micro-CT images were used to confirm ICO and SCO cannula placement. The stainless-steel tube length and skull cortical thickness at the cannula insertion site and whole skull were measured using RadiAnt DICOM Viewer software.

Rabbit Biotin-ASO extraction from brain tissue

Brain

Extraction (Standard)	
ASO amount (pmol)	Intensity
Blank	ND
10	ND
20	735.196
30	1728.359
50	4104.794
100	8327.685

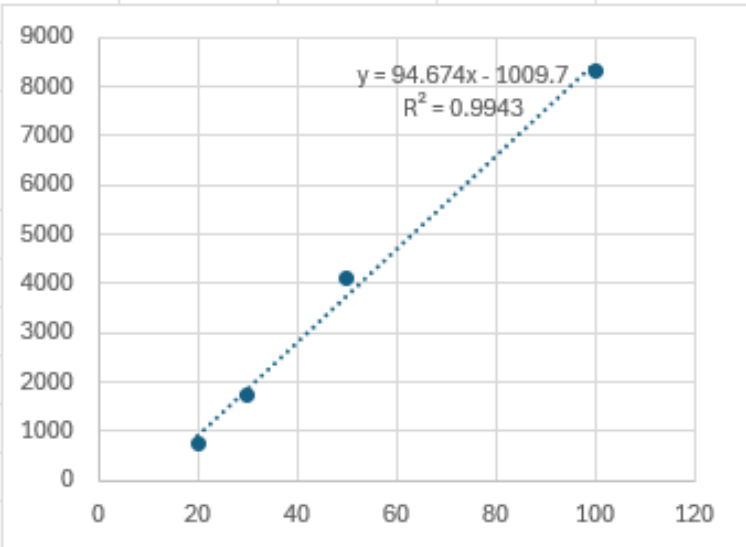

Mouse Biotin-ASO extraction from brain tissue

Brain

Extraction (Standard)	
ASO amount (pmol)	Intensity
Blank	ND
10	ND
20	772.832
30	1701.56
50	5064.815
100	10877.844

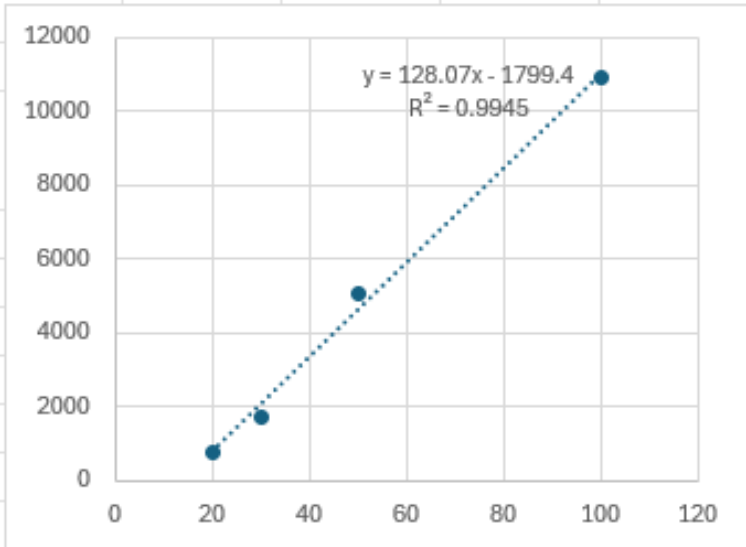

Mouse Biotin-ASO extraction from skull tissue

Skull

Extraction (Standard)	
ASO amount (pmol)	Intensity
Blank	ND
10	ND
20	1845.539
30	2929.388
50	5289.924
100	9977.409

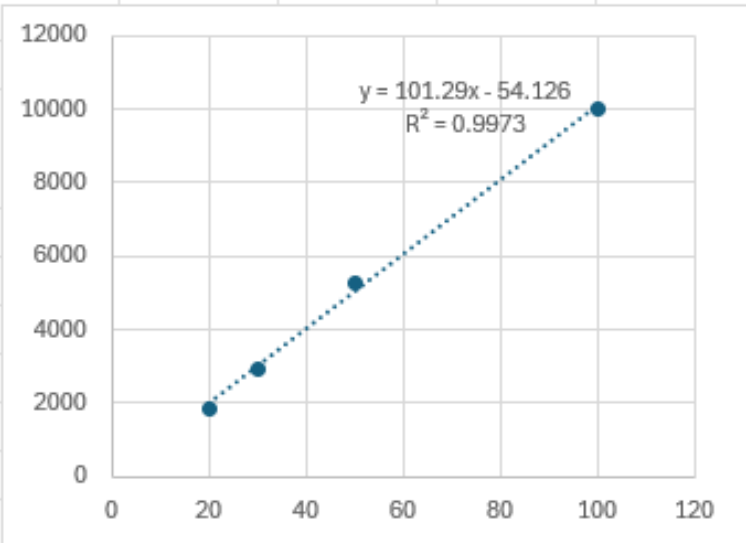

Mouse Biotin-ASO extraction from dura tissue

Dura

Extraction (Standard)	
ASO amount (pmol)	Intensity
Blank	ND
10	ND
20	1244.832
30	2023.731
50	3819.995
100	8530.723

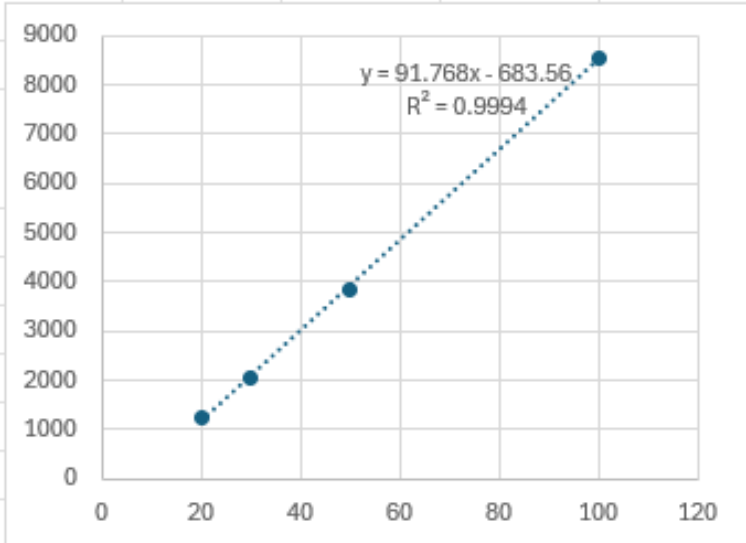

Figure S7. Matrix-matched standard curves for quantification of intact biotinylated ASO recovered from tissue homogenates.

Known amounts of biotinylated ASO were spiked into blank rabbit brain, mouse brain, mouse skull, or mouse dura homogenates and processed using the same streptavidin bead-based recovery procedure as experimental samples. Recovered ASO was analyzed by agarose gel electrophoresis, and band intensities were quantified by densitometry. Linear calibration curves were generated over the detectable range of 20–100 pmol. The 10-pmol standard was below the detection limit under the present assay conditions and was excluded from linear regression. Each calibration curve showed good linearity in the corresponding tissue matrix. Blank matrix samples showed no detectable ASO band at the expected size.

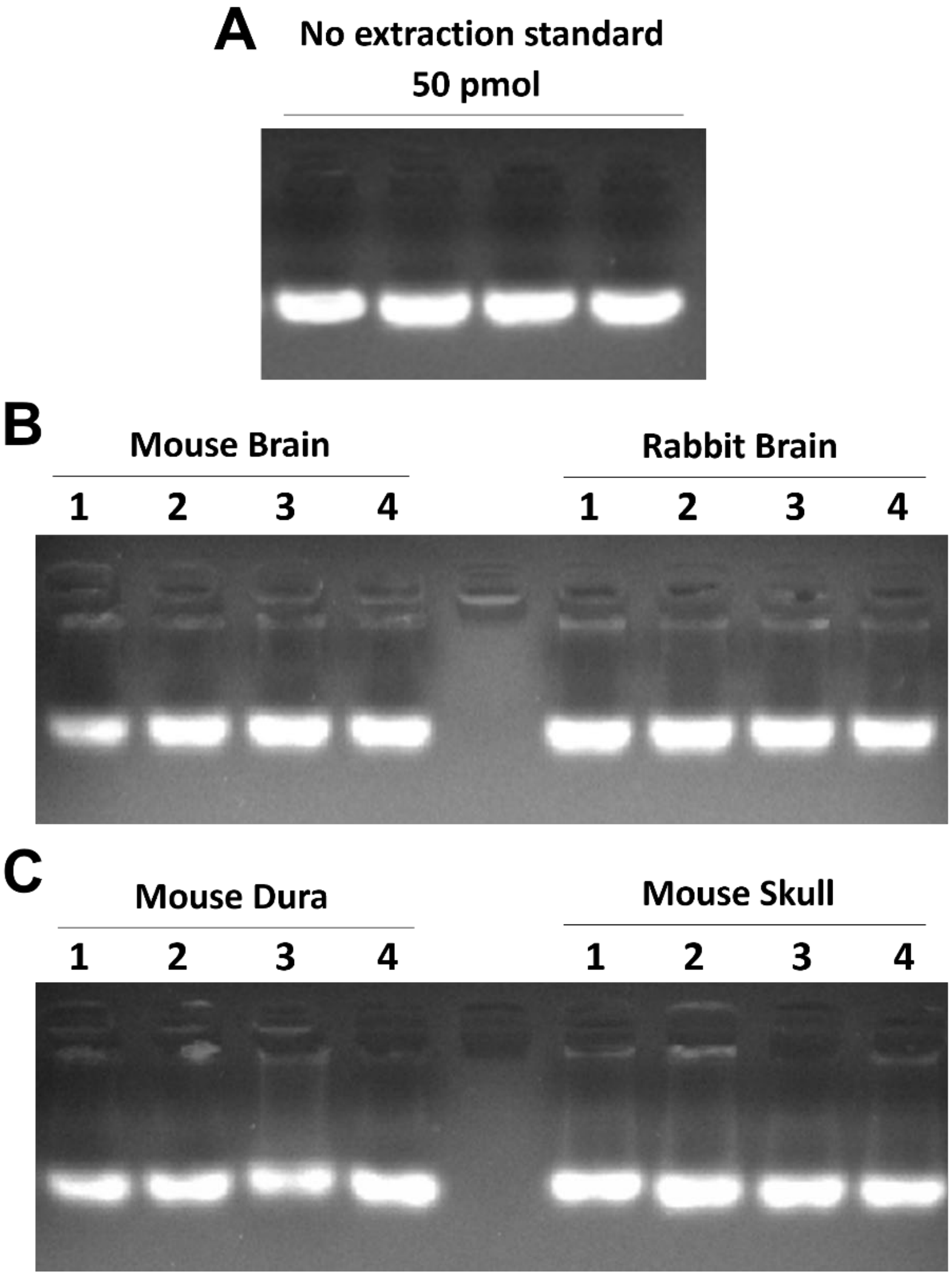

Figure S8. Matrix-matched extraction validation of intact biotinylated ASO.

(A) Non-extracted ASO standards used as recovery references. **(B, C)** Representative replicate gel images of 50-pmol biotinylated ASO spiked into mouse brain, rabbit brain, mouse dura, and mouse skull homogenates and recovered using the streptavidin bead-based extraction procedure. Intact ASO bands were detected in all tested matrices and used for recovery and intra-assay precision analysis.

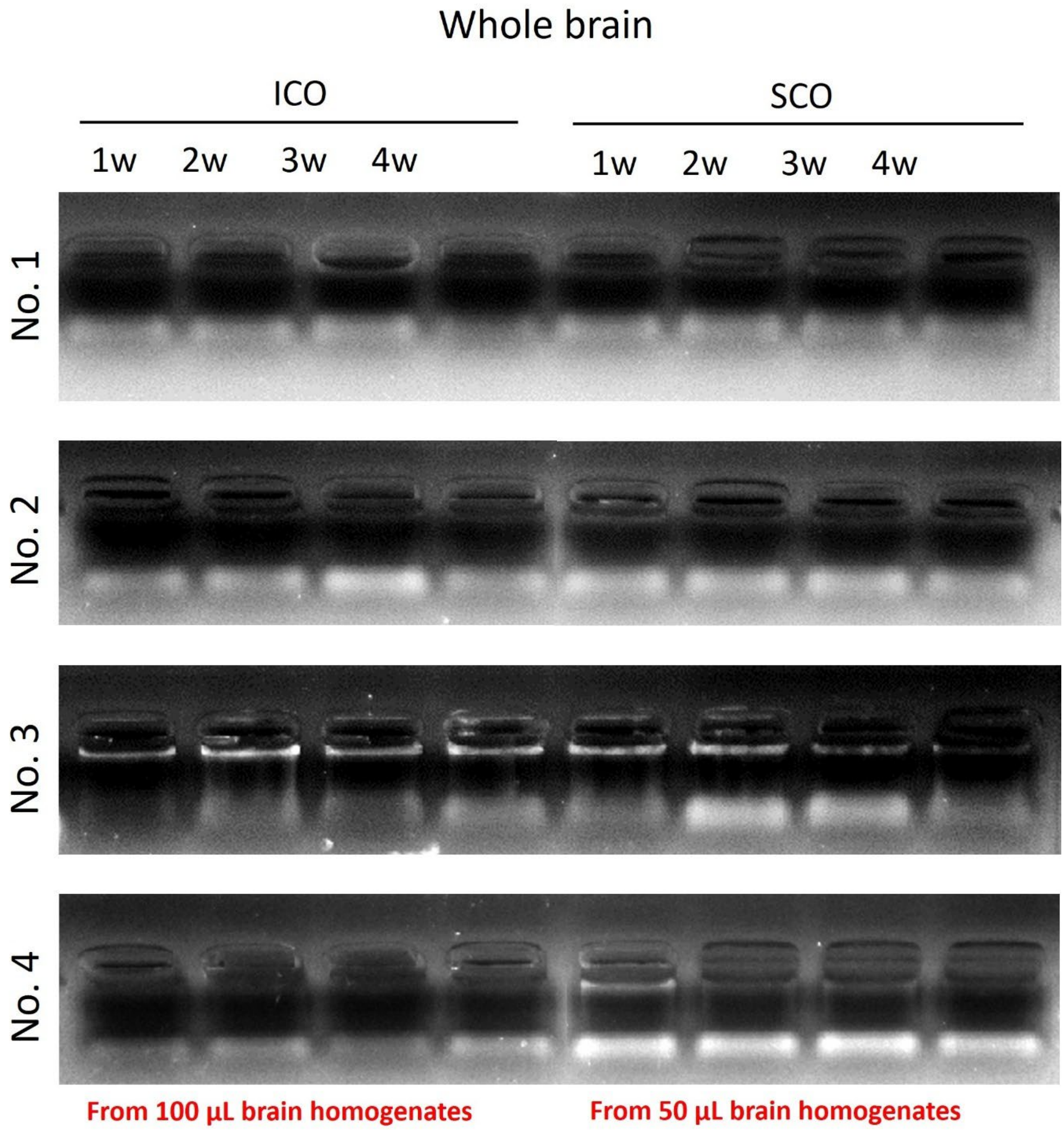

Figure S9. Gel electrophoresis analysis of intact ASO recovered from whole-brain homogenates of individual mice.

Gel band images show intact biotinylated scramble ASO recovered from brain homogenates of all mice after ICO or SCO. Samples were collected at weeks 1, 2, 3, and 4 after ICO or SCO, as indicated.

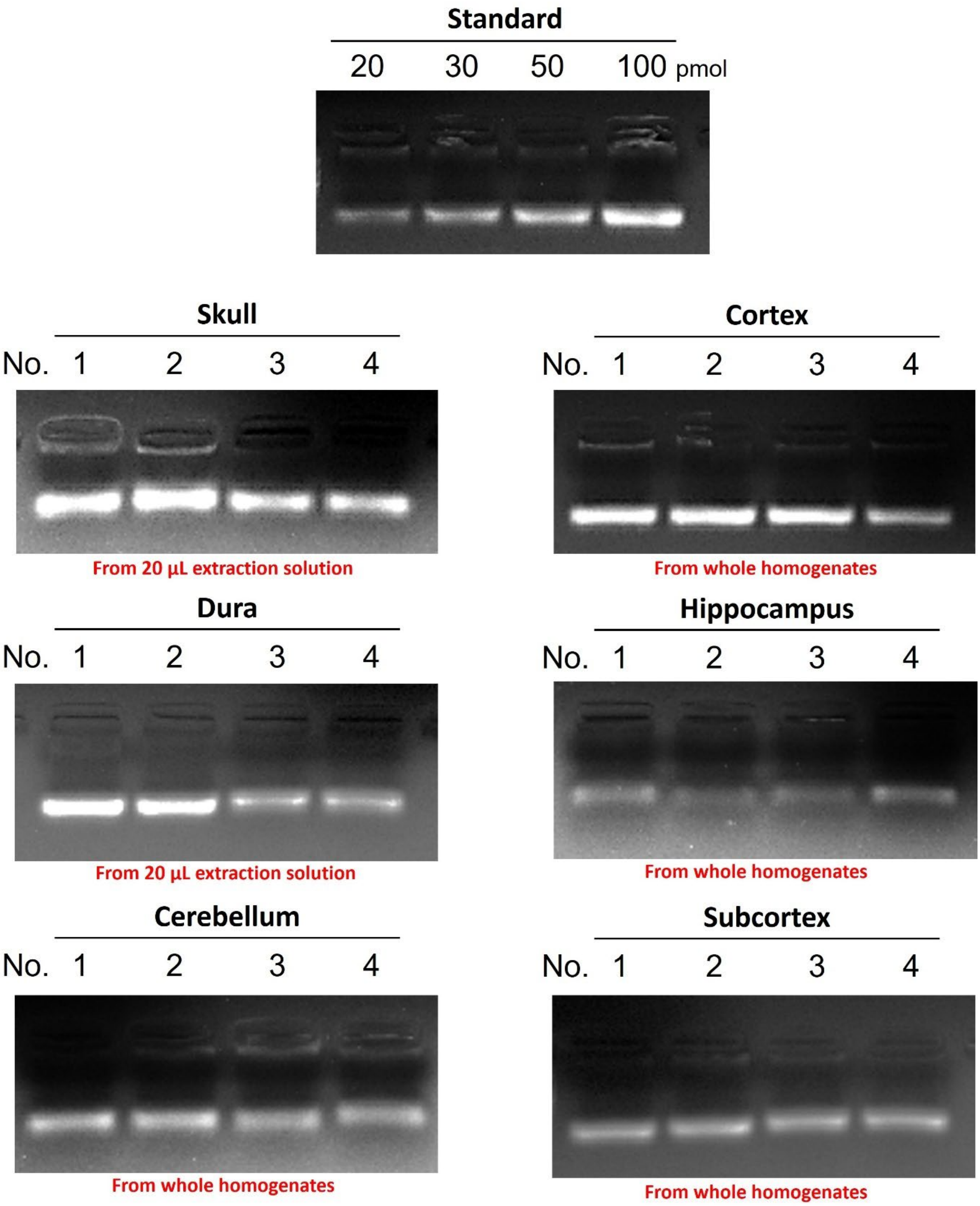

Figure S10. Gel electrophoresis analysis of intact ASO recovered from skull, dura, and regional brain homogenates after four-week ICO in mice.

Gel band images show intact biotinylated scramble ASO recovered from skull, dura, cortex, hippocampus, cerebellum, and subcortex homogenates of individual mice.

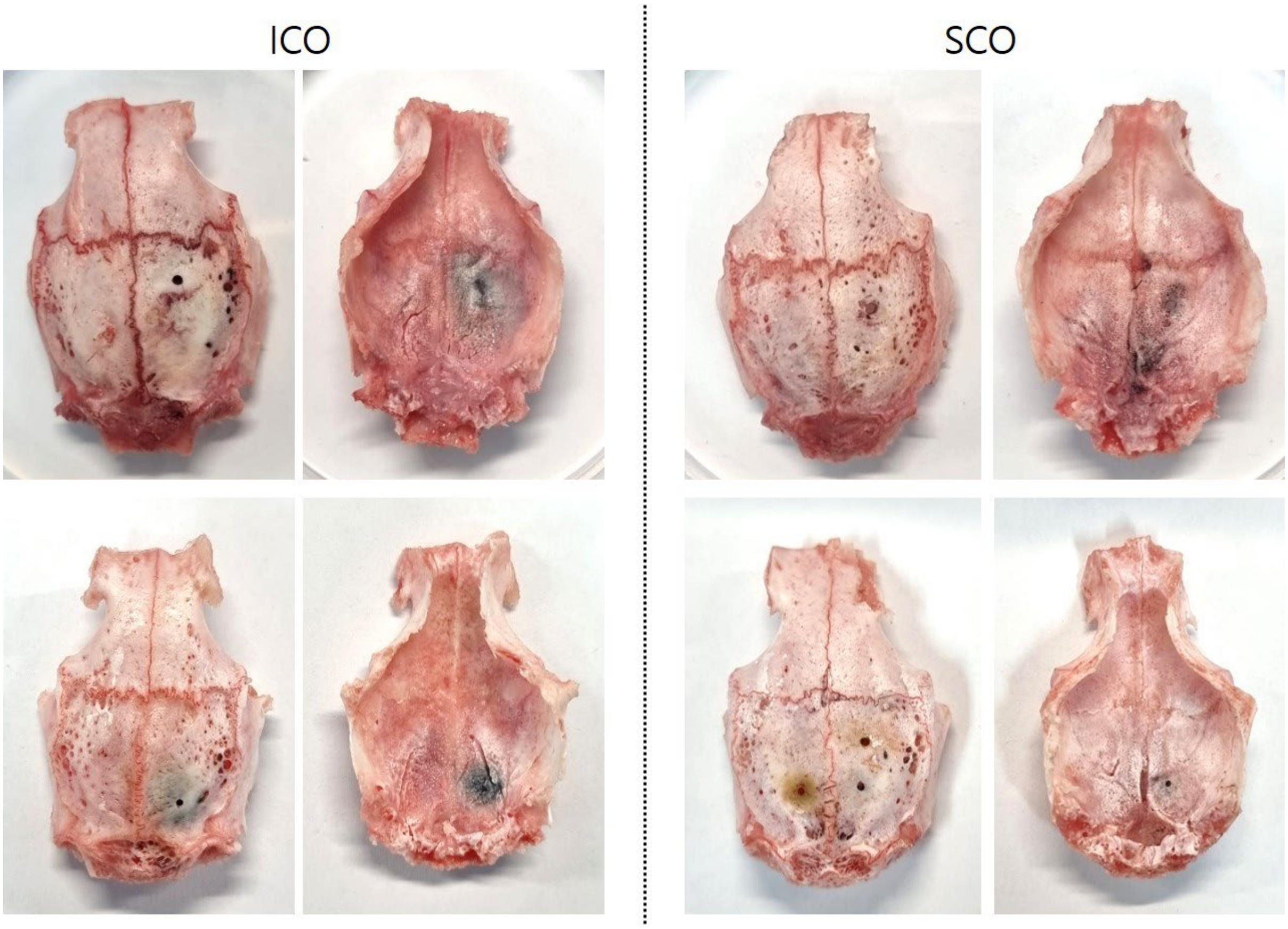

Figure S11. Photographs of dissected rabbit skulls after four-week ICO or SCO.

Black AuNP-associated regions were observed in the skull near the cannula insertion site. These signals were consistent with localization of AuNP within the skull diploic space between the outer and inner skull cortices.

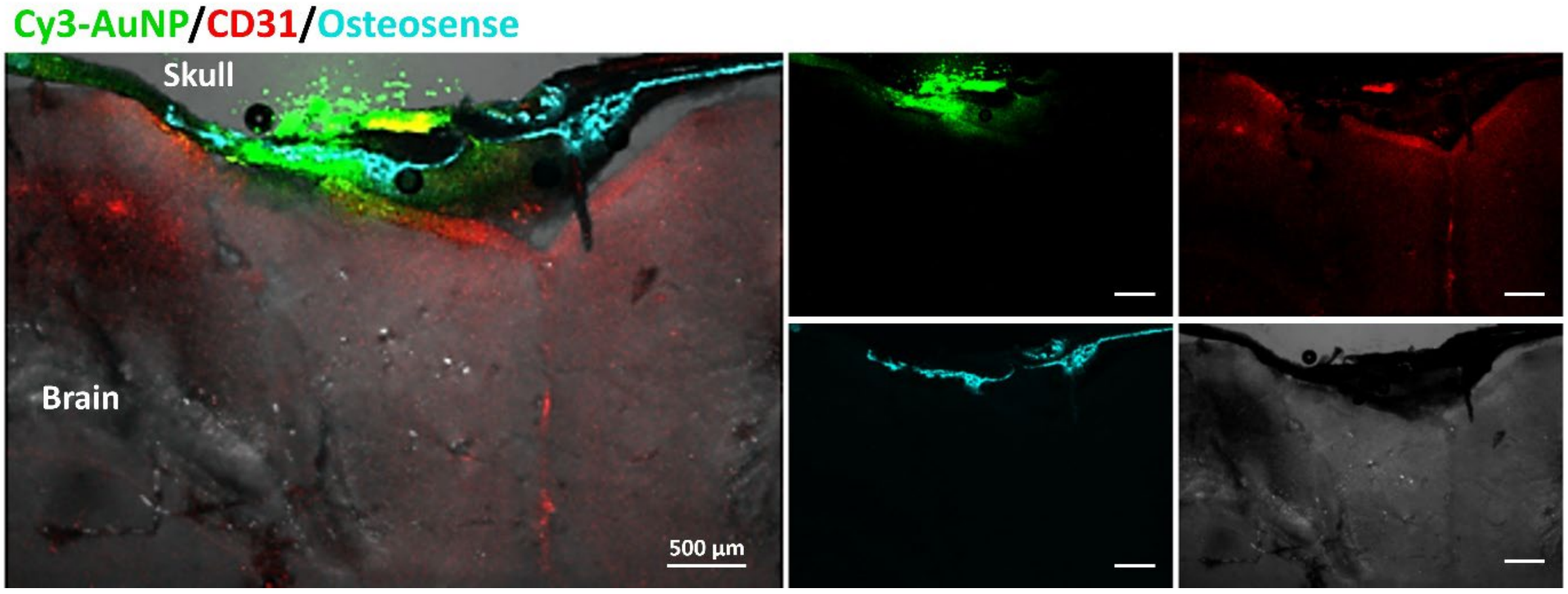

Figure S12. ***Ex vivo*** **confocal imaging of Cy3-AuNP localization in mouse coronal skull–brain sections after ICO.**

Cy3-AuNP signals were observed in the diploic space of the right ICO-side skull, whereas comparable signals were not apparent in the contralateral left skull under the imaging conditions used. Scale bar, 500 µm.

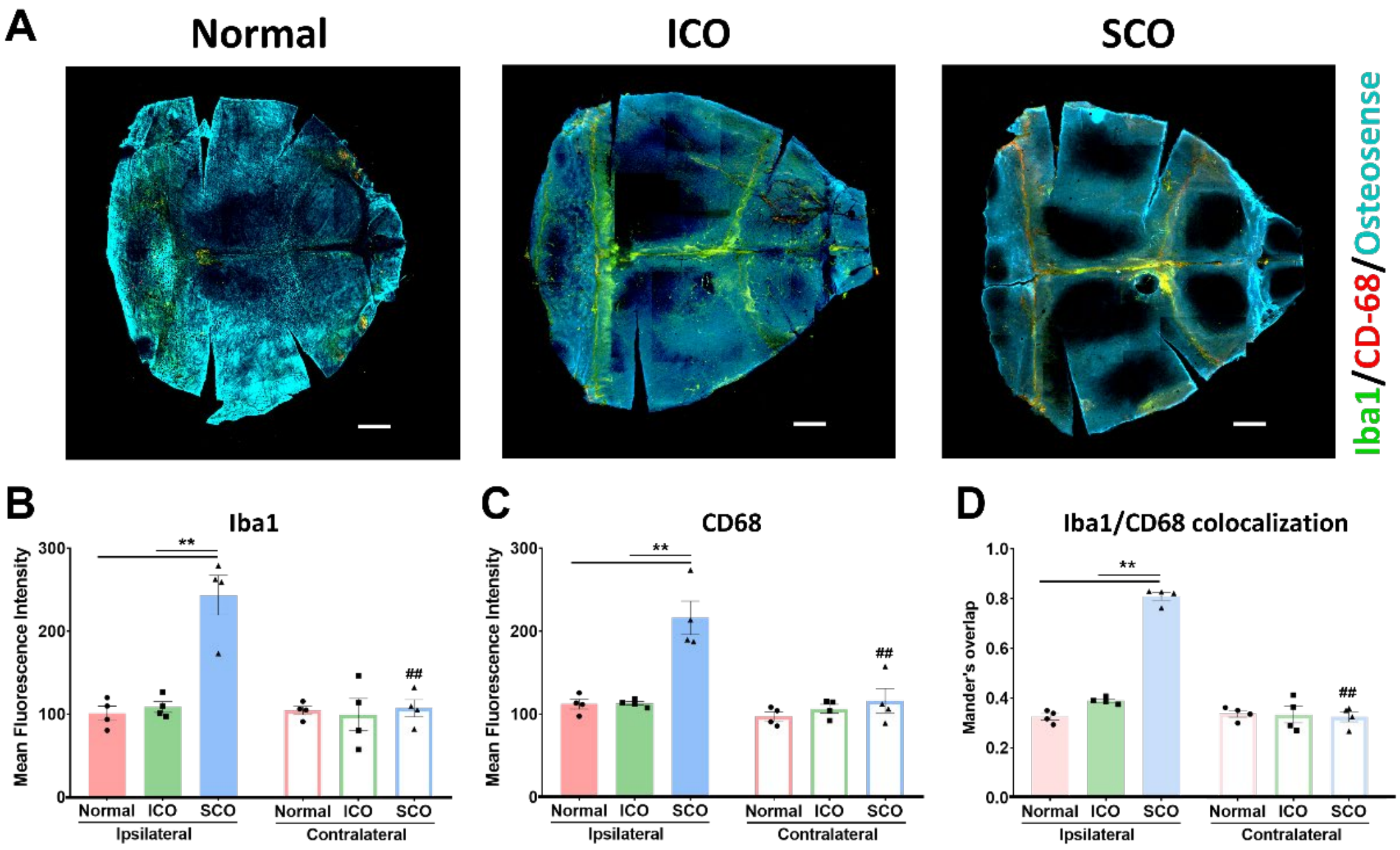

Figure S13. Quantitative skull whole-mount immunofluorescence after four-week ICO or SCO in mice.

(A) Representative maximum-intensity projections of decalcified and cleared mouse skull whole-mounts four weeks after ICO or SCO. Samples were immunostained for Iba1 and CD68 to visualize myeloid/microglia-associated inflammatory signals. OsteoSense was used to visualize mineralized skull bone. Scale bar, 1 mm. Quantification of **(B)** Iba1 and **(C)** CD68 mean fluorescence intensity in the ipsilateral skull region containing the drilling site (right parietal bone) and contralateral non-drilled skull region (left parietal bone). **(D)** Manders' overlap coefficient for Iba1/CD68 colocalization. Quantitative analysis showed stronger Iba1- and CD68-associated inflammatory signals and increased Iba1/CD68 colocalization at the ipsilateral skull region after SCO compared with ICO. Data are expressed as mean ± SEM (n = 4). Statistical significance within ipsilateral or contralateral regions among normal, ICO, and SCO groups is indicated by *$p < 0.05$ and **$p < 0.01$. Comparisons between ipsilateral and contralateral areas within the same group are denoted by #$p < 0.05$ and ##$p < 0.01$.

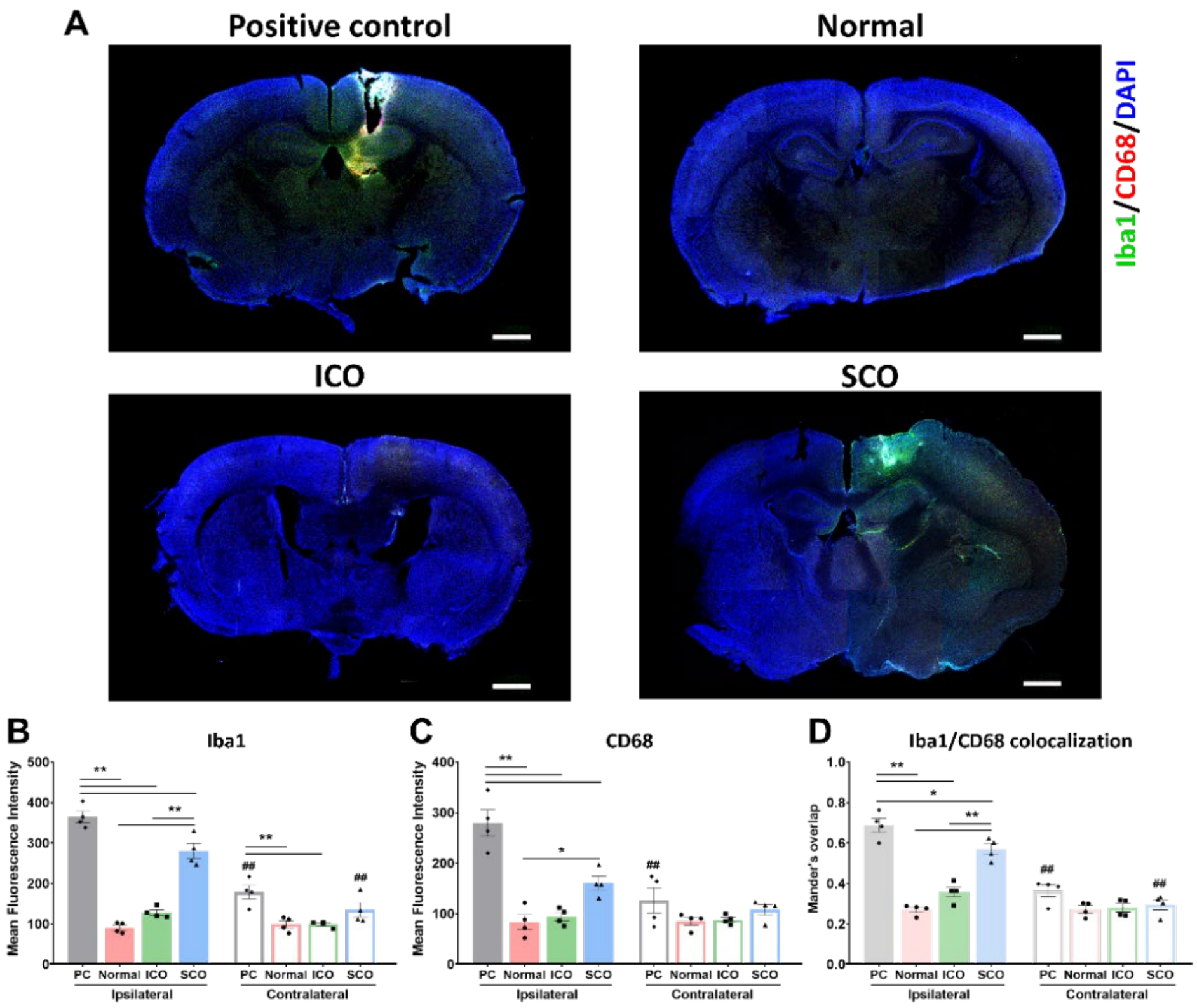

Figure S14. Quantitative brain-section immunofluorescence after four-week ICO or SCO in mice.

(A) Representative mouse brain sections imaged at 4× magnification four weeks after ICO or SCO. Sections were immunostained for Iba1 and CD68 to assess microglia/macrophage-associated inflammatory responses. Scale bar, 1 mm. Quantification of **(B)** Iba1 and **(C)** CD68 mean fluorescence intensity in ipsilateral and contralateral brain regions. **(D)** Manders' overlap coefficient for Iba1/CD68 colocalization. Quantitative analysis showed stronger Iba1- and CD68-associated inflammatory signals and increased Iba1/CD68 colocalization after SCO compared with ICO, particularly in ipsilateral brain regions. Data are expressed as mean ± SEM (n = 4). Statistical significance within ipsilateral or contralateral regions among positive control, normal, ICO, and SCO groups is indicated by $^*p < 0.05$ and $^{**}p < 0.01$. Comparisons between ipsilateral and contralateral areas within the same group are denoted by $\#p < 0.05$ and $\#\#p < 0.01$.

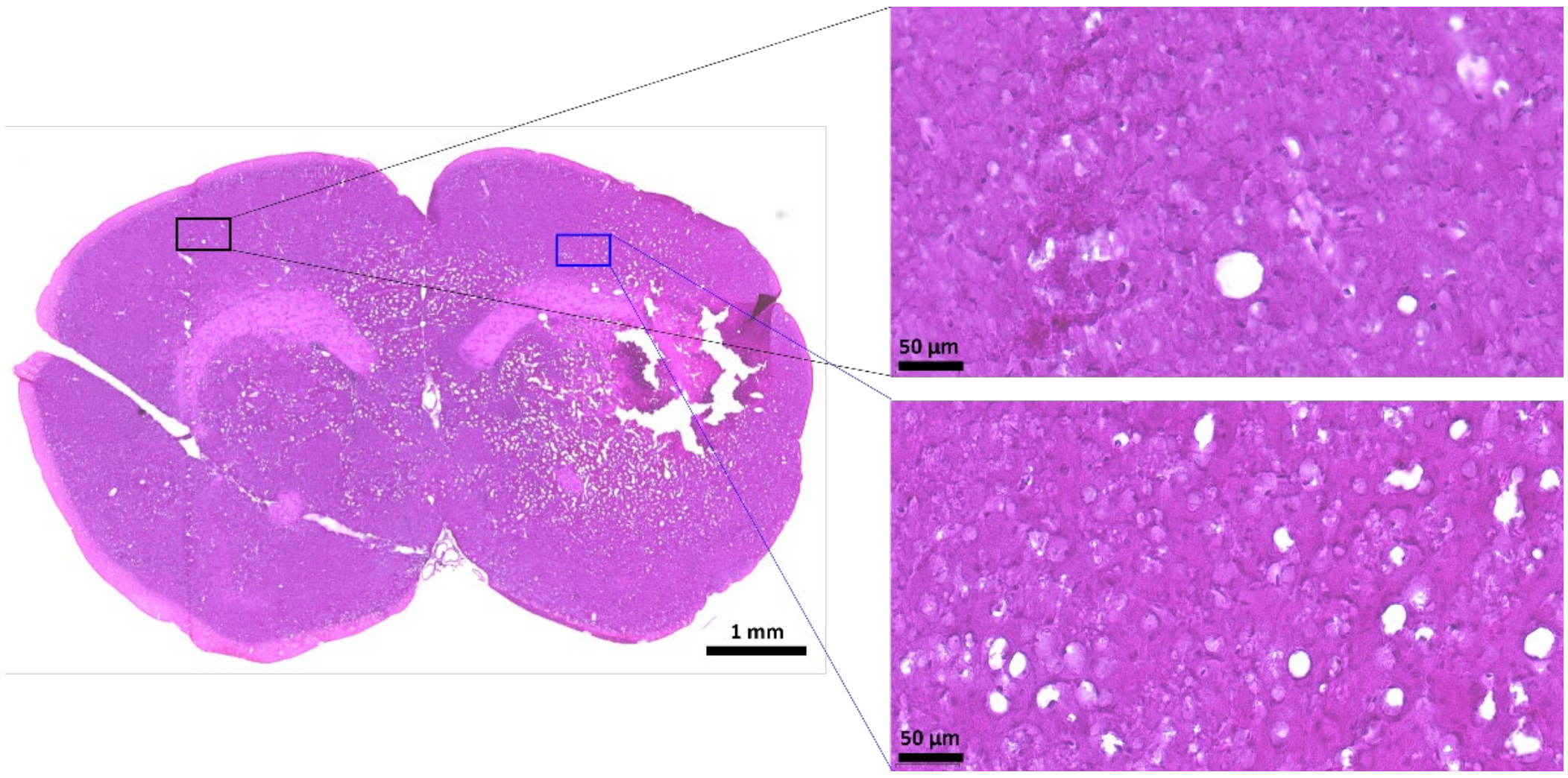

Figure S15. Representative H&E images supporting effective vascular blood removal after cardiac perfusion.

Representative H&E-stained brain section after cardiac perfusion with ice-cold PBS. Boxed cortical regions are shown at higher magnification. High-magnification images show no apparent accumulation of residual intravascular erythrocytes within cortical microvascular lumens, supporting effective reduction of vascular blood content before tissue-level analysis. Scale bars: 1 mm and 50 µm.

Table S1. Preoperative CT-based skull thickness at the target cannula insertion site in rabbits. The pooled mean ± SEM was 2.19 ± 0.04 mm across all rabbits with available measurements.

No.	Skull thickness of target position (mm)	
	**ICO**	**SCO**
1	2.36	2.04
2	2.23	2.26
3	2.17	2.09
4	2.33	2.05
5	2.15	
Mean ± SEM	2.19 ± 0.04	

Table S2. Quantitative micro-CT-based catheter placement analysis (n = 4 – 5, mean ± SEM).

Species	Mouse		Rabbit	
**Group**	ICO	SCO	ICO	SCO
**Intended target**	0.2 mm	Dural exposure	1 mm	Dural exposure
**Measured cannula tip depth**	0.193 ± 0.006 mm	0.380 ± 0.006 mm	1.034 ± 0.025 mm	1.994 ± 0.021 mm
**Inner skull cortex preserved**	yes	no	yes	no
**Full-skull thickness penetration**	no	yes	no	yes
**Placement success rate**	100% (4 / 4)	100% (4 / 4)	100% (5 / 5)	100% (4 / 4)

Data are presented as mean ± SEM. Measured cannula tip depth was defined as the distance from the outer skull surface at the drilling site to the cannula terminus. Placement success was defined as preservation of the inner skull cortex for ICO and full-thickness skull penetration with dural exposure for SCO.

Table S3. Validation of matrix-matched ASO recovery and gel densitometry assay (n=4, mean ± SEM).

Matrix	Rabbit brain	Mouse brain	Mouse dura	Mouse skull
**Calibration linear range**	20 – 100 pmol	20 – 100 pmol	20 – 100 pmol	20 – 100 pmol
**Calibration R^2**	0.9943	0.9945	0.9994	0.9973
**QC amount**	50 pmol	50 pmol	50 pmol	50 pmol
**n**	4	4	4	4
**Recovery (%)**	92.77 ± 2.56	87.72 ± 4.30	88.90 ± 3.69	94.05 ± 2.44
**Intra-assay precision (%CV)**	5.52	9.81	8.31	5.19
**Lowest detectable standard**	20 pmol	20 pmol	20 pmol	20 pmol

Recovery and intra-assay precision were assessed using four independent matrix-matched extraction replicates at 50 pmol. Calibration linearity was evaluated using matrix-matched standard curves over the detectable range.

Table S4. Concentrations of model agents in plasma, skull, dura, and regional brain tissues at week 4 after ICO in mice (n = 4, mean ± SEM). PTX concentrations were quantified by LC-MS/MS. ASO concentrations were quantified from gel band intensities of intact biotinylated ASO. Total gold concentrations were quantified by ICP-MS and used as a surrogate for AuNP-associated exposure. ND, not detected.

	PTX (ng/g)	ASO (pmol/g)	AuNP (ppb)
Plasma	0.26 ± 0.03	ND	9.59 ± 0.94
Skull	529.35 ± 100.79	10018.08 ± 821.77	6644.32 ± 920.78
Dura	138.07 ± 25.98	5435 ± 403.49	1459.21 ± 154.25
Cortex	5.64 ± 0.74	301.73 ± 33.57	113.75 ± 10.24
Hippocampus	4.36 ± 0.06	339.29 ± 24.89	96.17 ± 7.64
Cerebellum	5.81 ± 0.27	301.78 ± 15.90	104.36 ± 5.64
Subcortex	4.82 ± 0.32	280.87 ± 34.26	99.06 ± 7.18

Table S5. Hematologic indices in rabbit models at 4 weeks post-ICO or -SCO (n = 4 – 5, mean ± SEM). Control values represent preoperative baseline samples collected from four randomly selected animals before surgery.

Parameters (units) [a]	Control	ICO	SCO	Standard Range [b]
WBC ($x10^3$cells/$\mu\ell$)	6.77 ± 0.42	6.01 ± 0.21	7.07 ± 0.50	5.5 - 12.5
RBC ($x10^6$cells/$\mu\ell$)	6.63 ± 0.17	5.98 ± 0.20	6.52 ± 0.08	5.46 - 7.94
HGB (g/dL)	13.25 ± 0.32	12.80 ± 0.53	13.78 ± 0.19	10.4 - 17.4
HCT (%)	43.13 ± 0.78	40.78 ± 1.36	44.58 ± 0.96	36 - 50
MCV (fL)	65.18 ± 0.98	67.06 ± 0.64	68.03 ± 0.60	61 - 70
MCH (pg)	19.98 ± 0.17	20.92 ± 0.54	20.98 ± 0.15	19 - 26
MCHC (g/dL)	30.70 ± 0.55	31.24 ± 0.56	30.98 ± 0.41	30 - 37
RDW (%)	13.50 ± 0.33	12.06 ± 0.17	12.30 ± 0.30	11 - 16
PLT ($x10^3$ cells/$\mu\ell$)	455.00 ± 59.62	426.60 ± 44.97	404.00 ± 29.00	304 - 656
MPV(fL)	7.53 ± 0.22	7.04 ± 0.35	7.30 ± 0.45	5.2 - 9.6
Neutrophils (%)	24.88 ± 3.36	24.40 ± 2.61	29.00 ± 4.78	38 - 54
Lymphocytes (%)	66.33 ± 3.03	63.48 ± 3.27	63.10 ± 4.66	28 - 50
Monocytes (%)	3.43 ± 0.41	3.56 ± 0.52	3.33 ± 0.59	4 - 12
Eosinophils (%)	1.78 ± 0.24	1.80 ± 0.23	2.05 ± 0.43	0.5 - 3.5
Basophils (%)	5.50 ± 1.14	5.42 ± 0.72	5.23 ± 0.67	2.5 - 7.5
Large unstained cells (%)	0.60 ± 0.20	1.14 ± 0.57	1.05 ± 0.36	-

[a] WBC: white blood cell count; HGB: hemoglobin; MCH: mean corpuscular hemoglobin; RBC: red blood cell count; RDW: red cell diameter width; MCHC: mean corpuscular hemoglobin concentration; HCT: hematocrit; MCV: mean corpuscular volume; MPV: mean platelet volume; PLT: platelets.

[b] Baseline blood profiles of New Zealand rabbits based on existing literature [1, 2].

Literature-based reference intervals are provided for contextual comparison and may vary depending on strain, age, sex, sampling conditions, and analytical platform.

Table S6. Evaluation of biochemical markers in rabbits blood at 4 weeks post-ICO or -SCO (n = 4 – 5, mean ± SEM). Control values represent preoperative baseline samples collected from four randomly selected animals before surgery.

Parameters (units) [a]	Control (Normal)	ICO	SCO	Standard Range [b]
AST (U/L)	65.6 ± 10.54	50.5 ± 8.18	56.73 ± 9.91	35 - 130
ALT(U/L)	95.4 ± 9.30	79.5 ± 7.67	70.13 ± 5.96	45 - 80
ALP (U/L)	88.9 ± 7.09	64.9 ± 7.21	71.33 ± 7.51	12 - 96
Albumin (g/dL)	2.32 ± 0.02	2.23 ± 0.03	2.26 ± 0.07	2.7 - 5.0
BUN (mg/dL)	16.93 ± 0.99	16.50 ± 1.28	17.71 ± 1.43	20 - 45
Creatinine (mg/dL)	1.08 ± 0.05	1.15 ± 0.05	1.19 ± 0.03	0.5 - 2.5

[a] ALP: alkaline phosphatase; AST: aspartate aminotransferase; ALT: alanine aminotransferase; BUN: blood urea nitrogen.

[b] Standard biochemical values of New Zealand rabbits reported in previous studies [2, 3].

Literature-based reference intervals are provided for contextual comparison and may vary depending on strain, age, sex, sampling conditions, and analytical platform.

Table S7. Hematologic indices in mouse models at 4 weeks post-ICO or -SCO (n = 4, mean ± SEM). Control values represent preoperative baseline samples collected from four randomly selected animals before surgery.

	Groups			
**Parameters (units)** [a]	**Control**	**ICO**	**SCO**	**Standard Range** [b]
WBC (x10^3cells/μℓ)	6.05 ± 0.11	6.23 ± 0.18	6.84 ± 0.33	3.48 – 14.03
RBC (x10^6cells/μℓ)	7.39 ± 0.26	7.12 ± 0.47	7.24 ± 0.36	6.93 – 12.24
HGB (g/dL)	13.70 ± 0.20	13.30 ± 0.47	13.33 ± 0.27	12.6 – 20.5
HCT (%)	43.75 ± 0.77	41.10 ± 1.52	43.40 ± 1.96	42.1 – 68.3
MCV (fL)	60.28 ± 2.08	61.58 ± 2.13	62.68 ± 3.03	50.7 – 64.4
MCH (pg)	19.45 ± 0.47	20.38 ± 0.57	17.50 ± 2.77	13.2 – 17.6
MCHC (g/dL)	29.83 ± 0.83	31.65 ± 0.86	30.43 ± 0.72	23.3 – 32.7
RDW (%)	13.30 ± 0.33	13.05 ± 0.17	12.13 ± 0.40	16.9 – 23.4
PLT (x10^3 cells/μℓ)	546.25 ± 23.50	493.25 ± 36.29	476.75 ± 70.07	420 – 1698
MPV(fL)	7.35 ± 0.23	7.20 ± 0.13	7.63 ± 0.22	4.6 – 5.9
Neutrophils (%)	22.53 ± 0.87	25.43 ± 1.05	28.65 ± 2.02	9.86 – 39.11
Lymphocytes (%)	67.15 ± 1.54	64.20 ± 1.43	59.10 ± 2.55	48.81 – 83.19
Monocytes (%)	3.05 ± 0.19	3.18 ± 0.24	4.18 ± 0.17	0.11 – 4.91
Eosinophils (%)	1.78 ± 0.13	1.83 ± 0.09	2.85 ± 0.06	3.29 – 12.48
Basophils (%)	5.50 ± 1.14	5.38 ± 0.93	5.23 ± 0.67	0.00 – 1.84

[a] WBC: white blood cell count; HGB: hemoglobin; MCH: mean corpuscular hemoglobin; RBC: red blood cell count; RDW: red cell diameter width; MCHC: mean corpuscular hemoglobin concentration; HCT: hematocrit; MCV: mean corpuscular volume; MPV: mean platelet volume; PLT: platelets.

[b] Baseline blood profiles of Balb/c mice based on existing literature [4].

Literature-based reference intervals are provided for contextual comparison and may vary depending on strain, age, sex, sampling conditions, and analytical platform.

Table S8. Evaluation of biochemical markers in mice blood at 4 weeks post-ICO or -SCO (n = 4, mean ± SEM). Control values represent preoperative baseline samples collected from four randomly selected animals before surgery.

	Groups			
**Parameters (units)** [a]	**Control**	**ICO**	**SCO**	**Standard Range** [b]
AST (U/L)	159.98 ± 6.60	161.25 ± 8.05	164.23 ± 6.61	55 - 352
ALT(U/L)	64.25 ± 3.29	65.78 ± 2.52	65.28 ± 2.56	41 – 131
ALP (U/L)	259.48 ± 24.16	225.55 ± 21.43	255.83 ± 32.10	118 – 433
Albumin (g/dL)	2.81 ± 0.17	2.67 ± 0.18	2.80 ± 0.25	2.7 – 4.6
BUN (mg/dL)	18.72 ± 1.26	17.28 ± 1.54	17.87 ± 0.87	7 – 26
Creatinine (mg/dL)	0.27 ± 0.03	0.30 ± 0.05	0.28 ± 0.04	0.2 – 0.4

[a] ALP: alkaline phosphatase; AST: aspartate aminotransferase; ALT: alanine aminotransferase; BUN: blood urea nitrogen.

[b] Standard biochemical values of Balb/c mice reported in previous studies [4].

Literature-based reference intervals are provided for contextual comparison and may vary depending on strain, age, sex, sampling conditions, and analytical platform.

Table S9. Body weight changes in rabbits after four-week ICO or SCO (n = 4 – 5, mean ± SEM).

No.	Body weight (kg)			
	ICO		SCO	
	Day 0	Week 4	Day 0	Week 4
1	3.40	3.27	3.41	3.36
2	3.30	3.15	3.44	3.22
3	3.32	3.35	3.61	3.73
4	3.51	3.59	3.55	3.62
5	3.60	3.65		
Mean ± sem	3.43 ± 0.06	3.40 ± 0.09	3.50 ± 0.05	3.48 ± 0.12

Table S10. Body weight changes in mice after four-week ICO or SCO. (n = 4, mean ± SEM).

No.	Body weight (g)			
	ICO		SCO	
	Day 0	Week 4	Day 0	Week 4
1	19.2	19.6	19.5	19.4
2	18.9	19.0	19.7	20.0
3	19.7	19.6	19.7	19.5
4	20.0	20.1	20.2	19.8
Mean ± sem	19.45 ± 0.25	19.58 ± 0.23	19.78 ± 0.15	19.68 ± 0.14

Table S11. Effect sizes and 95% confidence intervals (CI) for skull whole-mount immunofluorescence.

Marker	Region	Comparison	Mean difference	95% CI	Hedges' g	Adjusted p value
**Iba1 MFI**	Ipsi lateral	Normal vs ICO	0.1408	-37.26 to 37.54	0.2354	>0.9999
		Normal vs SCO	-115.8	-153.2 to -78.42	4.1659	<0.0001
		ICO vs SCO	-116.0	-153.4 to -78.56	4.1400	<0.0001
	Contra lateral	Normal vs ICO	3.878	-33.52 to 41.27	0.0485	0.9994
		Normal vs SCO	2.169	-35.23 to 39.57	0.0955	>0.9999
		ICO vs SCO	-1.709	-39.11 to 35.69	0.0822	>0.9999
**IL-1β MFI**	Ipsi lateral	Normal vs ICO	-6.923	-47.72 to 33.87	0.5626	0.9937
		Normal vs SCO	-184.8	-225.6 to -144.0	7.1902	<0.0001
		ICO vs SCO	-177.9	-218.6 to -137.1	5.8310	<0.0001
	Contra lateral	Normal vs ICO	3.980	-36.81 to 44.77	-0.3927	0.9995
		Normal vs SCO	-12.68	-53.47 to 28.12	1.0256	0.9160
		ICO vs SCO	-16.66	-57.45 to 24.14	1.2695	0.7826
**Iba1/ IL-1β colocalization**	Ipsi lateral	Normal vs ICO	-0.07738	-0.1951 to 0.04037	2.1756	0.3357
		Normal vs SCO	-0.5194	-0.6642 to -0.3745	9.6966	<0.0001
		ICO vs SCO	-0.4420	-0.5597 to -0.3243	19.8946	<0.0001
**Iba1 MFI**	Ipsi lateral	Normal vs ICO	-7.605	-71.11 to 55.90	0.6113	0.9988
		Normal vs SCO	-142.5	-206.0 to -79.00	3.0080	<0.0001
		ICO vs SCO	-134.9	-198.4 to -71.40	2.9163	<0.0001
	Contra lateral	Normal vs ICO	4.929	-58.58 to 68.44	0.4626	0.9999
		Normal vs SCO	-2.698	-66.21 to 60.81	1.0838	>0.9999
		ICO vs SCO	-7.628	-71.13 to 55.88	0.0692	0.9988
**CD68 MFI**	Ipsi lateral	Normal vs ICO	-0.9942	-49.98 to 48.00	0.4947	>0.9999
		Normal vs SCO	-104.1	-153.1 to -55.09	3.2931	<0.0001
		ICO vs SCO	6.007	-42.98 to 55.00	3.2438	<0.0001
	Contra lateral	Normal vs ICO	-9.235	-58.23 to 39.76	0.2274	0.9898
		Normal vs SCO	-18.98	-67.97 to 30.01	0.6313	0.8164
		ICO vs SCO	-9.741	-58.73 to 39.25	0.5280	0.9870
**Iba1/ CD68 colocalization**	Ipsi lateral	Normal vs ICO	-0.06507	-0.1503 to 0.02016	2.9232	0.1990
		Normal vs SCO	-0.4851	-0.5863 to -0.3839	14.0299	<0.0001
		ICO vs SCO	-0.4200	-0.5052 to -0.3348	12.4176	<0.0001

Data were calculated using animal-level mean values after averaging 4 ROIs per animal. Mean difference and 95% CI were obtained from multiple-comparison analysis. Effect size was calculated as Hedges' g. MFI, mean fluorescence intensity.

Table S12. Effect sizes and 95% confidence intervals for brain-section immunofluorescence.

Marker	Region	Comparison	Mean difference	95% CI	Hedges' g	Adjusted p value
Iba1 MFI	Ipsi lateral	Normal vs ICO	-32.95	-84.18 to 18.29	1.4246	0.4253
		Normal vs SCO	-96.10	-147.3 to -44.87	5.0081	<0.0001
		ICO vs SCO	-63.16	-114.4 to -11.92	3.7837	0.0086
	Contra lateral	Normal vs ICO	-15.78	-67.02 to 35.45	0.3531	0.9666
		Normal vs SCO	-38.10	-89.33 to 13.14	1.0994	0.2579
		ICO vs SCO	-22.32	-73.55 to 28.92	0.8785	0.8286
IL-1β MFI	Ipsi lateral	Normal vs ICO	-11.97	-73.22 to 49.29	0.6931	0.9977
		Normal vs SCO	-106.9	-168.1 to -45.60	5.6559	0.0001
		ICO vs SCO	-94.89	-156.2 to -33.63	5.1223	0.0007
	Contra lateral	Normal vs ICO	-1.973	-63.23 to 59.28	0.2723	>0.9999
		Normal vs SCO	-14.49	-75.75 to 46.76	1.5653	0.9925
		ICO vs SCO	-12.52	-73.78 to 48.74	2.1753	0.9969
Iba1/ IL-1β colocalization	Ipsi lateral	Normal vs ICO	-0.06030	-0.1525 to 0.03189	2.1078	0.4047
		Normal vs SCO	-0.3232	-0.4154 to -0.2310	7.5999	<0.0001
		ICO vs SCO	-0.2629	-0.3551 to -0.1707	6.5707	<0.0001
Iba1 MFI	Ipsi lateral	Normal vs ICO	-37.21	-97.36 to 22.95	2.4708	0.4730
		Normal vs SCO	-189.4	-249.6 to -129.2	4.7936	<0.0001
		ICO vs SCO	-152.2	-212.3 to -92.03	3.7907	<0.0001
	Contra lateral	Normal vs ICO	-0.08582	-60.24 to 60.07	-0.6803	>0.9999
		Normal vs SCO	-35.72	-95.88 to 24.44	0.7636	0.5227
		ICO vs SCO	-35.63	-95.79 to 24.53	1.5552	0.5256
CD68 MFI	Ipsi lateral	Normal vs ICO	-10.69	-84.22 to 62.85	0.1111	0.9997
		Normal vs SCO	-77.50	-151.0 to -3.971	1.9990	0.0338
		ICO vs SCO	-66.82	-140.3 to 6.714	2.0977	0.0941
	Contra lateral	Normal vs ICO	-2.758	-76.29 to 70.77	0.4892	>0.9999
		Normal vs SCO	-22.80	-96.33 to 50.73	0.8730	0.9654
		ICO vs SCO	-20.04	-93.57 to 53.49	0.6653	0.9829
Iba1/ CD68 colocalization	Ipsi lateral	Normal vs ICO	-0.08840	-0.2054 to 0.02859	1.6121	0.2414
		Normal vs SCO	-0.2997	-0.4167 to -0.1827	5.4628	<0.0001
		ICO vs SCO	-0.2113	-0.3283 to -0.09429	3.0533	<0.0001

Data were calculated using animal-level mean values after averaging 4 ROIs per animal. Mean difference and 95% CI were obtained from multiple-comparison analysis. Effect size was calculated as Hedges' g. MFI, mean fluorescence intensity.

Supplementary material references

1. Moore DM, Zimmerman K, Smith SA. Hematological Assessment in Pet Rabbits: Blood Sample Collection and Blood Cell Identification. Clin Lab Med. 2015; 35: 617-27.
2. Black DM, Gilardi KV, Hamilton LP, Williams E, Williams DF, Kelly PA, et al. Hematologic and biochemistry reference values for the endangered riparian brush rabbit (Sylvilagus bachmani riparius). J Wildl Dis. 2009; 45: 491-6.
3. Melillo A. Rabbit Clinical Pathology. J Exot Pet Med. 2007; 16: 135-145.
4. Silva-Santana G, Bax JC, Fernandes DCS, Bacellar DTL, Hooper C, Dias A, et al. Clinical hematological and biochemical parameters in Swiss, BALB/c, C57BL/6 and B6D2F1 Mus musculus. Anim Models Exp Med. 2020; 3: 304-15.
